# Comprehensive analysis of m6A regulators and relationship with tumor microenvironment, immunotherapy strategies in colorectal adenocarcinoma

**DOI:** 10.1186/s12863-023-01149-y

**Published:** 2023-08-11

**Authors:** Jian Ji, Shichao Liu, Yongyuan Liang, Guixi Zheng

**Affiliations:** 1 Department of Clinical Laboratory, Shandong Province, Qilu Hospital of Shandong University, Jinan, 250012 People’s Republic of China; 2 Department of Clinical Laboratory, Shandong Province, Qilu Hospital, Cheeloo College of Medicine, Shandong University, Jinan, 250014 People’s Republic of China

**Keywords:** N6-methyladenosine, Colon adenocarcinoma, Prognosis, Tumor microenvironment, Immunotherapy

## Abstract

**Background:**

The N6-methyladenosine (m6A) RNA modification is the most prevalent and abundant type found in eukaryotic cells. It plays a crucial role in the initiation and progression of cancers. In this study, we aimed to comprehensively investigate the landscape of m6A regulators and their association with tumor microenvironment (TME), immunotherapeutic strategies in colon adenocarcinoma (COAD).

**Results:**

The differential expression, mutation, CNV frequency and prognostic value of 27 m6A regulators were systematically analyzed in COAD. Patients were classified into two clusters based on m6A regulators through consistent clustering analysis, with cluster A showing significant survival benefits. Most of the m6A regulators were negatively correlated with immune cells, except for WTAP, IGF2BP3, FTO, ALKBH5, which showed a positive correlation. We developed an m6A scoring system to calculate the m6Ascore for each patient. Patients with a high-m6Ascore had a better outcome, with the AUC of 0.775. An independent cohort of 416 COAD patients acquired from GSE38832 database was used to validate the prognosis prediction ability of m6Ascore. Moreover, the m6Ascore was negatively correlated with infiltration of anti-tumor immune cells. Additionally, patients with a high-m6Ascore responded better to anti-PD1 and anti-CTLA4 therapies, and those with MSI-H had a higher m6Ascore. Finally, we investigated the value of m6Ascore in predicting the response of patients to 15 commonly used drugs.

**Conclusions:**

We comprehensively analyzed m6A regulators in COAD, including RNA expression, CNV changes, mutations and their correlation with TME. Our results showed that the m6A scoring system had significant predictive power for the prognosis of COAD patients, potentially leading to new personalized immunotherapy strategies.

**Supplementary Information:**

The online version contains supplementary material available at 10.1186/s12863-023-01149-y.

## Background

Colorectal cancer (CRC) is a highly prevalent primary digestive tract tumors and is the third most commonly diagnosed cancer with the second highest mortality rate [1]. Colon adenocarcinoma (COAD), the main type of CRC, arises from adenomatous lesions [2]. Unfortunately, the 5-year survival rate is less than 15% when diagnosed at a late stage. Therefore, to enhance the clinical outcome of COAD patients, there is an urgent need for more effective prognostic biomarkers and therapeutic targets.

N6-methyladenosine (m6A) is a methylation modification of the sixth nitrogen (N) atom of adenine (A) that impacts RNA metabolic processes including splicing, transport, translation, and degradation [3]. As the most abundant and evolutionarily conserved modification of eukaryotic mRNA, the reversible methylation of m6A has a profound impact on the regulation of gene expression [4–6]. Abnormal expression of m6A regulators has been shown to play a crucial role in the occurrence and development of human cancers [7]. Additionally, their role as prognostic markers has also been reported in various cancers such as gastric cancer [8], liver cancer [9], lung adenocarcinoma [10], pancreatic cancer [11], thyroid cancer [12]. With the in-depth understanding of tumor microenvironment (TME), alterations of immune cell subsets have become increasingly recognized for their clinical implications in the occurrence, metastasis and prognosis of cancers [13–15]. Notably, the alteration of m6A modification bas been found to promote tumor immune escape by affecting TME [16, 17]. Thus, it is imperative to investigate whether m6A regulators can serve as prognostic biomarkers for COAD patients and their association with immune regulation and TME formation.

In this study, we examined the differential expression of m6A regulators between COAD tumors and normal tissues, the frequency of copy number variation (CNV) changes and the mutations of these regulators. Using univariate-Cox regression analysis, we identified 12 m6A regulators associated with prognosis. Our clustering analysis identified two distinct m6A clusters, cluster A and B, with patients in cluster A having better outcomes. We also investigated the relationship between m6A regulators and immune cells infiltration. Then, the differentially expressed genes (DEGs) between two clusters were identified by R software, with 1,164 DEGs identified as survival associated genes. Using principal component analysis (PCA), we calculated the m6Ascore of individual patient and grouped them into high- and low-m6Ascore groups. Our findings indicated that the m6Ascore was significantly associated with survival status, clinical stage, and TNM stage of patients. Furthermore, we observed a negative correlation between the m6Ascore and the infiltration of most immune cells by Spearman analysis. Additionally, the immune score, stromal score and Estimation score of COAD patients were negatively correlated with the m6Ascore. Patients with MSI-H exhibited higher m6Ascores. We further investigated the relationship between m6Ascore and the effectiveness of 15 commonly used drugs in COAD patients by IC_50_ analysis. The overview of study was shown in Supplementary Figure 1.

## Materials & methods

### Gene datasets and clinical information data collection

RNA-seq transcriptome data, simple nucleotide variation data and clinical information of COAD patients were acquired from public TCGA database. The GSE40967 dataset contains 585 COAD samples and 574 patients had survival information, which was downloaded from the GEO database. FPKM values acquired from TCGA database were transformed into transcript per kilobase million (TPM) in order to integrated with GEO data. The copy number variation (CNV) data was downloaded from UCSC database (https://xena.ucsc.edu). A list of immune-related genes was obtained from import database (https://immport.niaid.nih.gov/). The clinical characteristics of patients in TCGA and GSE40967 datasets were summarized in Table 1.

**Table 1:** Characteristics of COAD patients

Characteristic		TCGA cohort		GSE40967 cohort	
		No.of patients	Percentage (%)	No.of patients	Percentage (%)
Age	< 60	122	26.99	153	26.15
	≥ 60	330	73.01	432	73.85
Gender	Male	238	52.65	322	55.04
	Female	214	47.35	263	44.96
Clinical stage	I	76	16.81	42	7.18
	II	178	39.39	271	46.32
	III	125	27.65	210	35.90
	IV	62	13.72	60	10.26
	Unknown	11	2.43	2	0.34
T stage	T1	10	2.21	16	2.74
	T2	77	17.04	49	8.38
	T3	308	68.14	379	64.78
	T4	57	12.61	119	20.34
	Unknown	0	0	22	3.76
N stage	N0	269	59.51	314	53.68
	N1	103	22.79	137	23.42
	N2	80	17.70	106	18.12
	Unknown	0	0	28	4.78
M stage	M0	334	73.89	499	85.30
	M1	62	13.72	61	10.43
	Mx	49	10.84	3	0.51
	Unknown	7	1.55	22	3.76
Survival status	Alive	364	80.53	395	67.52
	Dead	88	19.47	179	30.60
	Unknown	0	0	11	1.88

### Landscape of m6A regulators in COAD

The expression level, mutation, CNV frequency and prognostic value of 27 m6A regulators, including 10 writers (METTL3, CBL1, METTL14, METTL16, WTAP, VIRMA, RBM15, RBM15B, ZNF217 and ZC3H13), 15 readers (YTHDC1, YTHDC2, YTHDF1, YTHDF2, YTHDF3, EIF3B, HNRNPC, FMR1, HNRNPA2B1, LRPPRC, IGF2BP1, IGF2BP2, IGF2BP3, ELAVL1 and RBMX) and 2 erasers (FTO and ALKBH5) which have been reported in published papers [18–20] were comprehensively analyzed.

The differential expression level of m6A regulators between 472 COAD tumor and 41 non-tumor tissues from public TCGA database was analyzed by R package “Limma”. To explore the prognostic predictive value of 27 m6A regulators, univariate-Cox regression was used. Then K-M curve was plotted to demonstrate the predictive ability of each m6A regulator. A network was performed to visualize the high and low risk factors and the correlation among the m6A regulators.

### Consensus cluster classification based on m6A regulators

The consensus clustering algorithm was used to identify distinct m6A modification patterns based on the expression of m6A regulators. The clustering program was performed using R package “Consensus Cluster Plus”. The distribution of each m6A regulator in different m6A clusters and clinical characters was shown by Heatmap.

### Gene set variation analysis (GSVA)

R package “GSVA” was used to investigate the variation in biological processed between different m6A clusters. The DEGs between distinct clusters were identified by R package “limma”. The genes with the false discovery rate (FDR) < 0.05 and Log_2_ |(FC)|≥ 1.0 were considered as DEGs. The Gene Oncology (GO) set “c5.go.v7.4.symbols.gmt” and Kyoto Encyclopedia of Genes and Genomes (KEGG) set “c2.cp.kegg.v7.4.symbols.gmt” based on the DEGs were used for GSVA analysis.

### Immune cell infiltration analysis by single sample gene set enrichment analysis (ssGSEA)

To explore the relation of m6A cluster with infiltration of immune cells, the proportion of 23 immune cells from each COAD sample was calculated using ssGSEA method by R. The difference of ssGSEA score of each immune cell between distinct m6A clusters was displayed by boxplot.

### Construction of m6Ascore

The above mentioned DEGs were employed to perform survival analysis using univariate-Cox regression by R. The survival-associated DEGs were selected to perform PCA analysis by combine principal component 1 and principal component 2 which was run and visualized by R script shown in supplementary original data.

Then m6Ascore of individual COAD patient was calculated by a formula provided in previous study as $\mathrm{m}6\mathrm{Ascore}={\sum }\left({\mathrm{PC}1}_{i}+{\mathrm{PC}2}_{i}\right)$ [21]. COAD patients were divided into high- and low-m6Ascore groups according to the median of m6Ascore. K-M and ROC curves were performed to display the survival status of two groups. An independent cohort of 416 COAD patients acquired from GSE38832 database was used to validate the prognostic prediction ability of the m6Ascore.

### Evaluation the relationship of m6Ascore with TME

The correlation of m6Ascore and immune cells was displayed by R package “corrplot”. The m6Ascore in COAD patients of different clinical characteristics was also analyzed. Furthermore, the immune score, stromal score and ESTIMATE score were calculated using estimation of stromal and immune cells in malignant tumor tissues using expression (ESTIMATE) algorithm by R package “estimate” and compared in the high- and low-m6Ascore groups.

### The value of m6Ascore in predicting response of patients to chemotherapy, targeted therapy and immunotherapy treatments

The immunophenoscore (IPS) and MSS/MSI status of 462 COAD patients were downloaded from The Cancer Immunome Database (TCIA) (https://www.tcia.at/home). The different MSS/MSI-L/MSI-H status and curative effect of anti-CTLA-4 and anti-PD-1 in low- and high-m6Ascore groups were analyzed by R. To explore the value of m6Ascore in predicting response of patients to chemotherapy, targeted therapy and immunotherapy treatments, we analyzed the IC_50_ of 15 commonly used drugs of COAD patients. The differences of the IC_50_ between two groups were compared by the wilcoxon signed-rank test using R packages “pRRophetic”.

### Statistical analysis

Wilcoxon test was performed to analyze the differential expression of m6A regulators between tumor and non-tumor tissues, DEGs between low- and high-m6Ascore groups by R (version 4.1.0). The relationship between m6Ascore and immune cells was analyzed by Spearman’s correlation analysis. Univariate-cox regression analysis was used to identify the survival associated m6A regulators and DEGs. K-M method was used to analyze the difference in overall survival (OS) between the high- and low- m6Ascore groups. *P*-value less than 0.05 on both sides was considered as statistically significant difference.

## Results

### Differential expression of m6A regulators in patients with COAD

We analyzed the differential expression of 27 m6A regulators in COAD tumor tissues compared with non-tumor tissues. Among these m6A regulators, METTL3, CBLL1, METTL16, WTAP, VIRMA, RBM15, RBM15B, ZC3H13, YTHDC1, YTHDF1/2, EIF3B, HNRNPC, FMR1, HNRNPA2B1, LRPPRC, IGF2BP1/2/3, ELAVL1, RBMX and FTO showed higher expression in COAD tumor tissues, while the expression of ALKBH5 was lower. The other four regulators (METTL14, ZNF217, YTHDC2 and YTHDF3) showed no significant difference between the two groups (Supplementary Figure 2A).

### The CNV and mutation frequency analyses of m6A regulators

CNV refers to the gain or loss of segments of the genome. Numerous studies have highlighted the significance of CNV as a source of genetic variation in cancers. Detecting CNV is crucial for a comprehensive understanding of the genome’s plasticity and its potential role in contributing to diseases. In this study, we analyzed the CNV changes of m6A regulators and the detailed locations of these regulators on chromosomes, as well as the frequency of CNV gain or loss (Supplementary Figure 2B and C). Our results demonstrated that CNV changes were prevalent in COAD. Specially, the copy numbers of ZNF217, IGF2BP1/2/3, EIF3B, HNRNPA2B1, WTAP, FMR1 and FTO showed a high frequency of gain, whereas RBM15, YTHDF2, METTL14, YTHDC2 and RBM15B displayed more loss of copy number.

We created a plot to show the prevalence of mutations in the aforementioned m6A regulators in COAD. The results revealed that 130/399 (32.58%) patients had mutational m6A regulators and ZC3H13 was the gene with the highest mutation rate of 9% (Supplementary Figure 3A). Since then, we tried to investigate whether the expression of other regulators was associated with ZC3H13 mutation. We found that the expressions of ZNF217 and YTHDF1 were lower in the ZC3H13 mutation group as compared to the wildtype group. Conversely, the expressions of METTL3 and ALKBH5 were higher in the mutation group (Supplementary Figure 3B). The results suggested that the expression of ZNF217, YTHDF1, METTL3 and ALKBH5 was significantly correlated with ZC3H13 mutation which was worth to further study.

### Evaluation of the prognostic value of m6A regulators

In order to evaluate the prognostic value of m6A regulators, univariate cox regression and K-M analyses were performed in 1,026 COAD patients from TCGA and GSE40967 databases. As shown in Supplementary Figure 4, 12 out of 27 m6A regulators were associated with prognosis of patients. CBL1, IGF2BP1, LRPPRC, YTHDC2 and YTHDF1 were found to be positively related to survival, functioning as protective factors. On the contrary, seven genes (ALKBH5, HNRNPC, FTO, WTAP, HNRNPA2B1, METTL3 and ZC3H13) were negatively to survival and considered as risk factors. The interaction network of m6A regulators and their roles in predicting prognosis were visualized in Supplementary Figure 5.

### Clustering of COAD patients based on m6A regulators

**Fig. 1:**
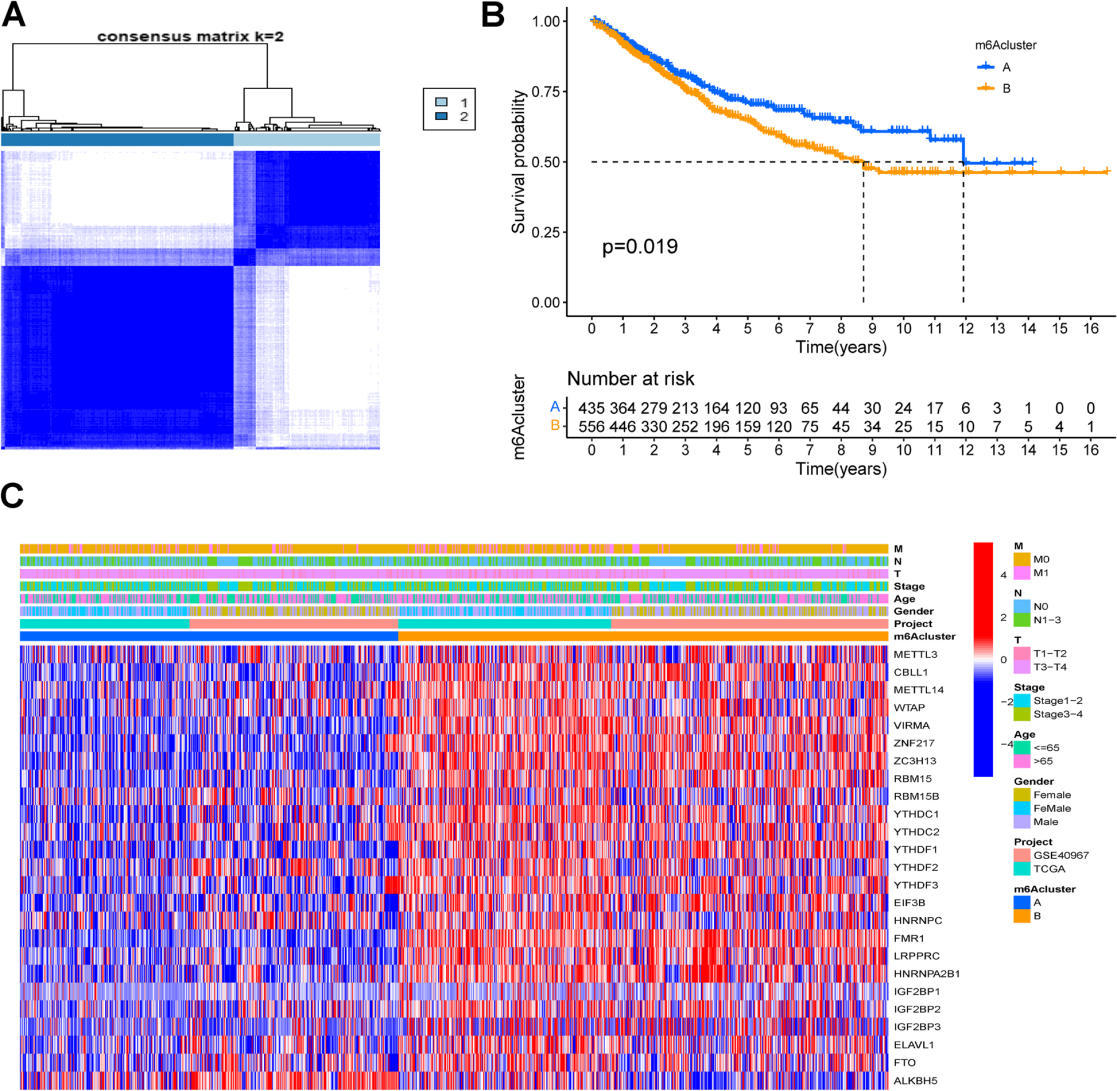
Survival of COAD patients in two clusters. **A** Consensus clustering heatmap in COAD (k = 2). **B** K-M curve of overall survival for COAD patients of two m6A clusters in TCGA and GSE40967 cohort. The patients in cluster A showed a significant better outcome than those in cluster B (*P* = 0.019). **C** Heatmap and clinicopathological characteristics of m6A regulators in the two clusters. All the m6A regulators except for ALKBH5 were upregulated in cluster B group

It was reported that m6A regulators were important in the classification of distinct clusters. In the study, COAD patients were classified into two clusters based on the expression level of m6A regulators (Fig. 1A). K-M curve indicated that patients in cluster A exhibited better outcomes than those in cluster B (Fig. 1B, *P* = 0.019). A heatmap was used to visualize the expression difference of m6A regulators between the two clusters (Fig. 1C). The results demonstrated a significant upregulation of ALKBH5 and downregulation of other m6A regulators in cluster A. No significant differences in age, gender, clinical stage, or TNM stages were observed between the two clusters.

### GO enrichment and KEGG pathway analyses

To investigate the biological and molecular mechanisms underlying the two clusters of COAD, we conducted GSVA analysis. GO results (supplementary Figure 6A) showed that several biological processes and molecular functions, such as vascular endothelial growth factor receptor signaling pathway, lymph angiogenesis, and lymph vessel development, were enriched in cluster B comparing to cluster A. KEGG results (supplementary Figure 6B) indicated that cluster A was found to be significantly enriched in butanoate metabolism, pyruvate metabolism, Huntington’s disease and glyoxylate and dicarboxylate metabolism, while cluster B was enriched in carcinogenic activation pathways, such as TGF-β signaling pathway, adherens junction, pathway in cancer, basal cell carcinoma, which might contribute to the poor prognosis of patients in cluster B.

### The relationship of m6A regulators with the infiltration of immune cells

**Fig. 2:**
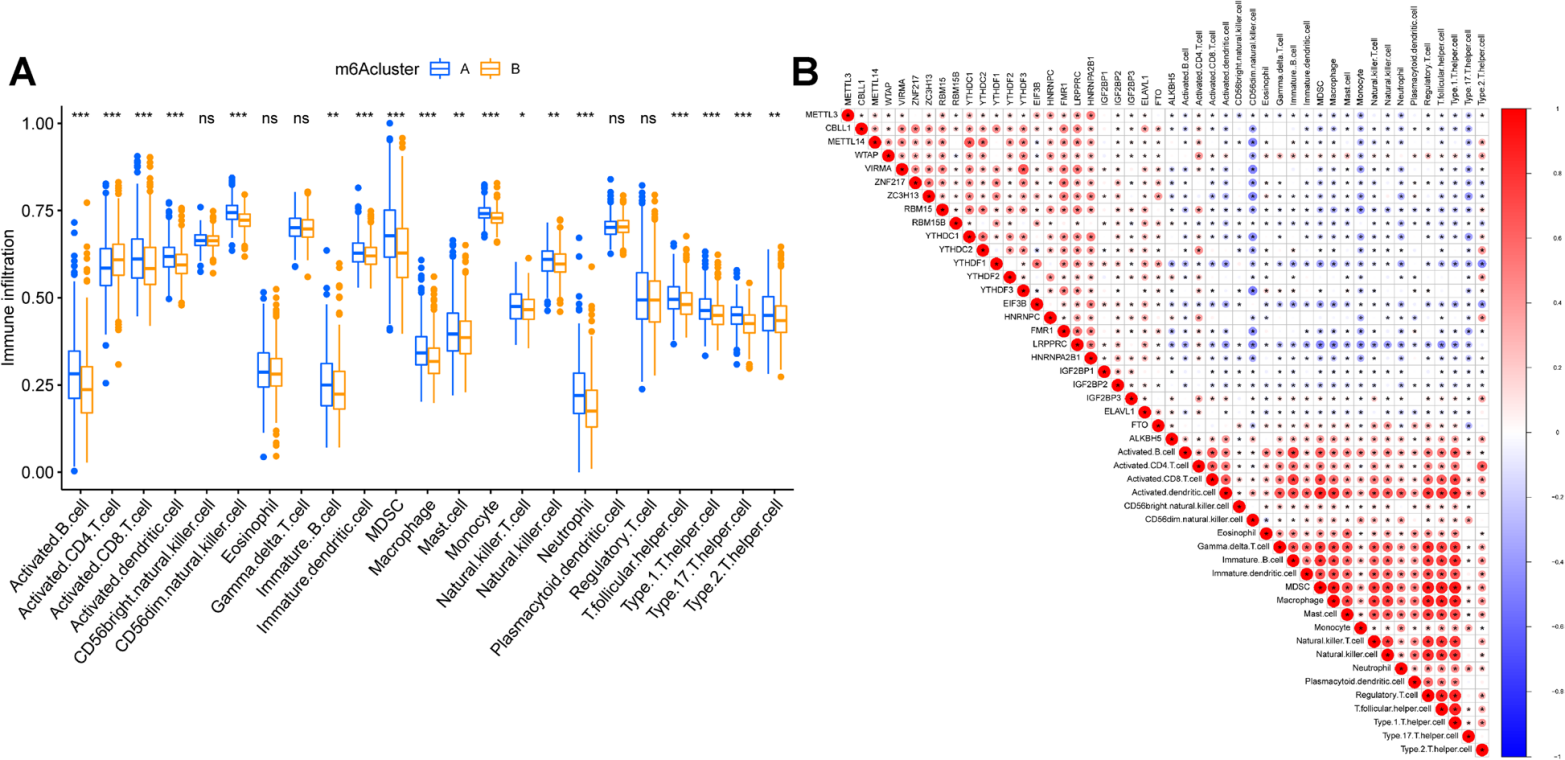
Proportion of immune cells and correlation between immune cells and m6A regulators. **A** The enrichment scores of 23 kinds of immune cells were compared between the two m6A clusters. The immune infiltration of most immune cells was higher in cluster A group. **B** Correlations between immune cells and m6A regulators analyzed by Spearman analysis. The significant negative and positive correlation were marked as blue * and red *

The enrichment scores of immune cells were estimated in the two clusters, revealing that the immune infiltration of various immune cells were higher in cluster A which might be one of the reasons that patients in cluster A had better outcomes (Fig. 2A).

Subsequent, Spearman’s correlation analysis was performed to investigate the association between each m6A regulator and immune cells. Our results indicated that the expressions of WTAP, IGF2BP3, FTO and ALKBH5 were positively correlated with enhanced immunocyte infiltration, while other m6A regulators including METTL3/14, CBLL1, VIRMA, RBM15, RBM15B, ZNF217, ZC3H13, YTHDC1, YTHDF1, EIF3B, FMR1 and LRPPRC, showed a negative association (Fig. 2B).

### Identification of m6A cluster associated DEGs

To investigate the genetic dysregulation, we observed differences between the two clusters and examined the transcriptional expression alterations related to m6A. A total of 8,154 DEGs were identified, of which 1,164 DEGs were considered as survival-associated genes and regarded as m6A cluster related genes (Supplementary Table 1). GO enrichment analysis indicated that these genes were involved in various biological processes, including transcription, phosphorylation, cell proliferation, migration, adhesion, cell cycle, apoptosis, angiogenesis and so on (Supplementary Figure 7A). KEGG pathway analysis demonstrated that the enriched pathways were metabolic pathways, pathways in cancer, MAPK signaling pathway, proteoglycans in cancer (Supplementary Figure 7B).

**Fig. 3:**
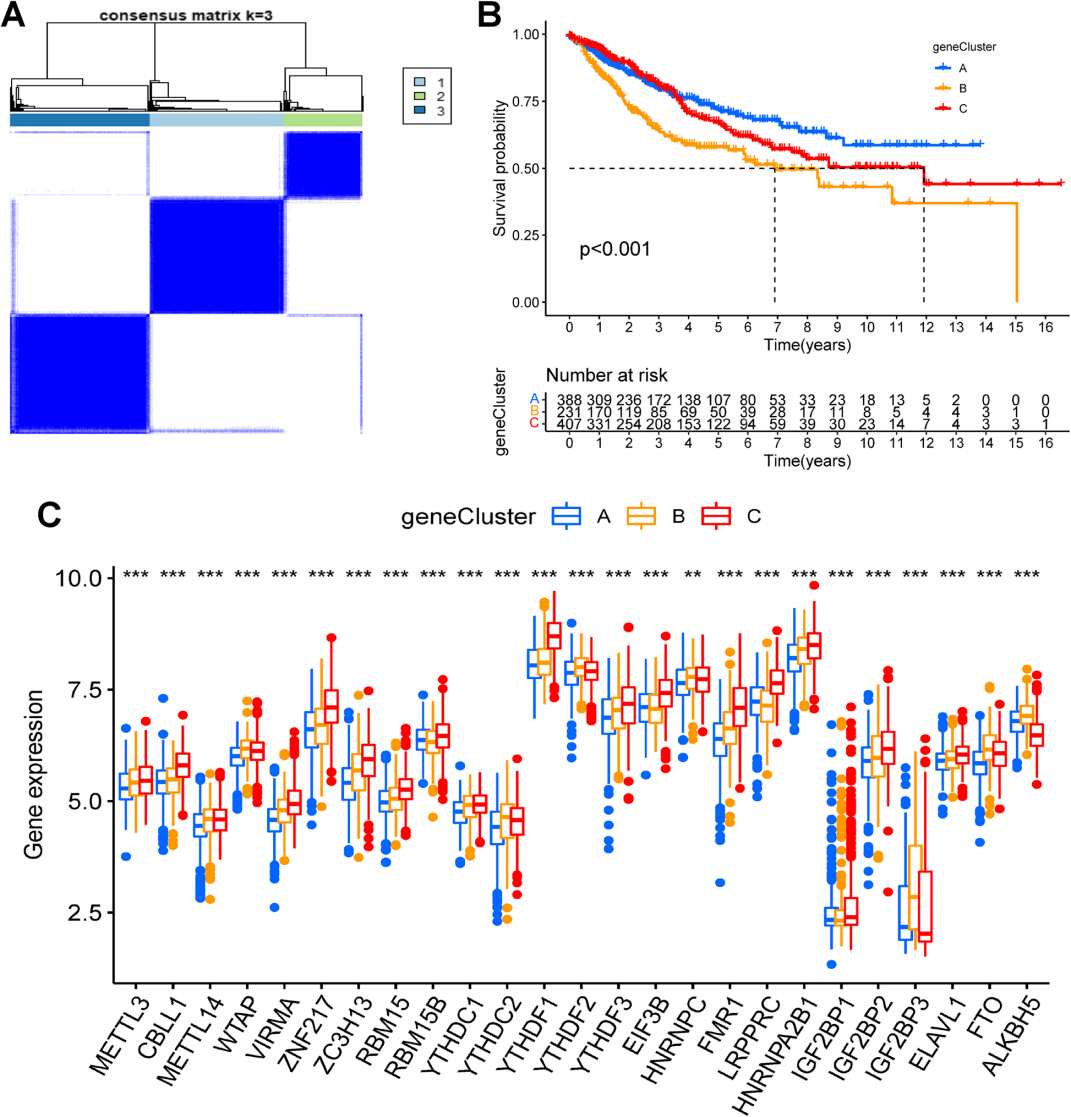
Gene clusters based on the m6A cluster related DEGs. **A** Consensus clustering heatmap in COAD (k = 3). **B** The K-M curves of three gene clusters based on m6A cluster-related genes. The gene cluster A showed a significant better outcome than cluster B and cluster C (*P* < 0.001). **C** The differential expression level of m6A regulators among three gene clusters in TCGA and GSE40967 cohorts

To explore the ability of clustering of the above DEGs, unsupervised consensus clustering analysis was performed. Patients were clearly divided into three gene clusters (A, B and C) **(**Fig. 3A). The survival analysis indicated a significant prognostic difference among the three gene clusters, with patients in gene cluster A having the best prognostic outcome, while patients in gene cluster B had the worst (Fig. 3B). The expressions of m6A regulators were also found to differ significantly among these three gene clusters **(**Fig. 3C**)**.

### m6Ascore is correlated with survival and clinical characteristics of patients

**Fig. 4:**
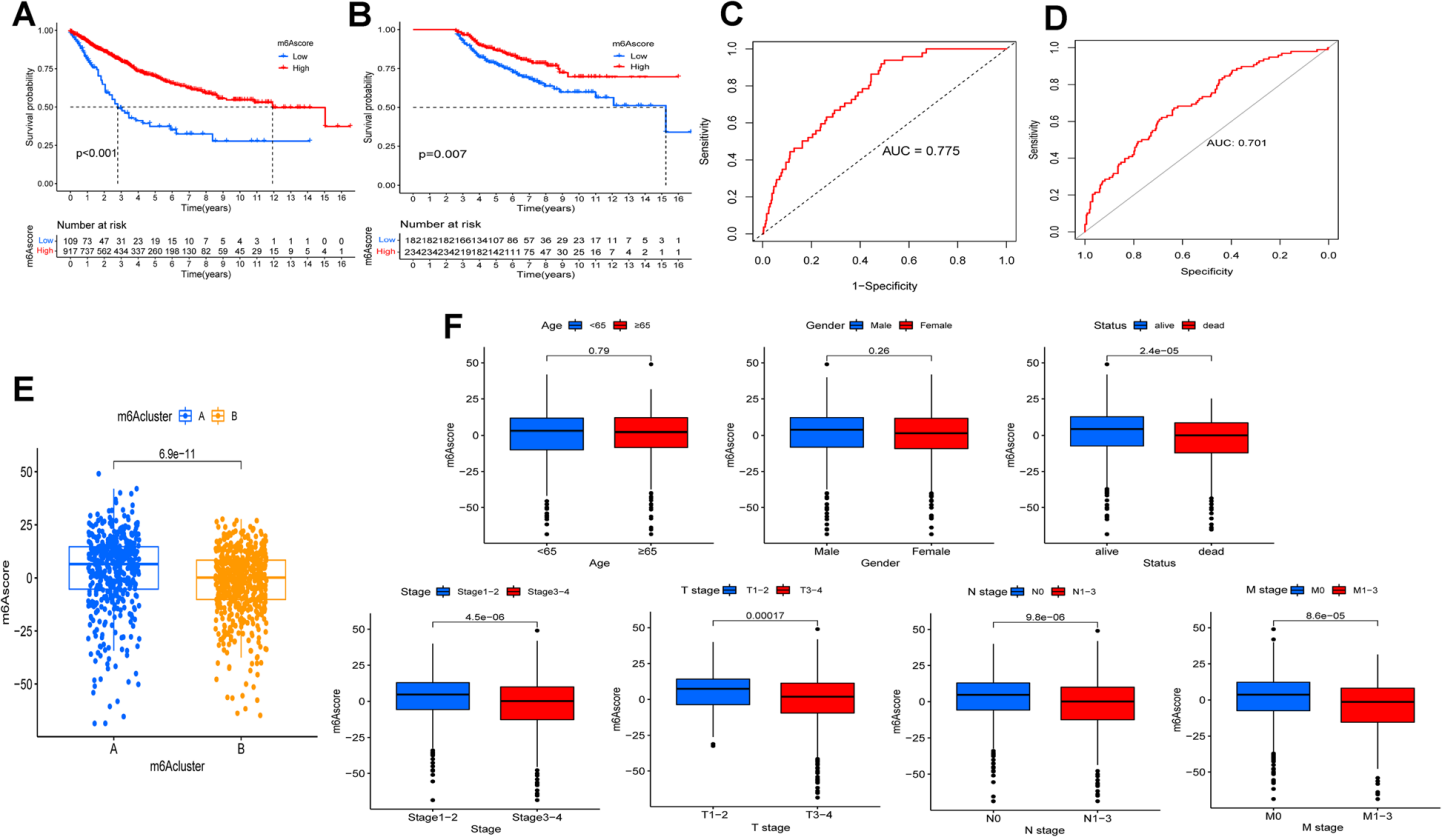
Construction of the m6Ascore and evaluation of the relationship between m6Ascore and clinical characteristics of COAD patients. **A** K-M curve of high- and low-m6Ascore groups. The patients in high-m6Ascore group had better outcome than those in low-m6Ascore group (*P* < 0.001). **B** K-M curve of an independent validation cohort (*P* = 0.007). **C** The ROC curve of the m6Ascore for predicting prognosis of COAD patients was plotted and the AUC was 0.775. **D** The AUC of the m6Ascore in validation cohort was 0.701. **E** The difference of m6Ascore was compared in the two m6A clusters. The m6Ascore was higher in cluster A (*P* = 6.9e-11). **F** The m6Ascore was correlated with patient’s survival status (*P* = 2.3e-05), clinical stage (*P* = 4.5e-06), T stage (*P* = 0.00017), N stage (*P* = 9.8e-06) and M stage (*P* = 8.6e-05). However, there was no significant correlation between m6Ascore and age (*P* = 0.79), gender (*P* = 0.26)

To assess the m6A pattern in individual COAD patient, we created a scoring system called m6Ascore by PCA analysis, which was base on the expression levels of m6A cluster-related DEGs. By categorizing patients into high- (917 cases) and low-m6Ascore groups (109 cases) according to the m6Ascore, we observed the patients in the high-m6Ascore group had better outcomes (*P* < 0.001, Fig. 4A) which was validated in an independent cohort of 416 COAD patients (*P* = 0.007, Fig. 4B). The area under the curve (AUC) was 0.775 in training cohort (Fig. 4C) and 0.701 (Fig. 4D) in validation cohort, suggesting that the m6Ascore was a reliable predictor of clinical outcomes for COAD patients.

We also compared the m6Ascore between cluster A and cluster B groups. The results showed that the m6Ascore was higher in patients of cluster A than those in cluster B, which was in agreement with m6A cluster survival analysis (*P* < 0.001, Fig. 4E). Subsequently, we explored the association between the m6Ascore and clinical features, the result of which indicated that the m6Ascore exhibited a significant correlation with survival status, clinical stage, TNM stage of patients, while no such correlation was observed with age and gender (Fig. 4F).

### The relationship of m6Ascore and TME

**Fig. 5:**
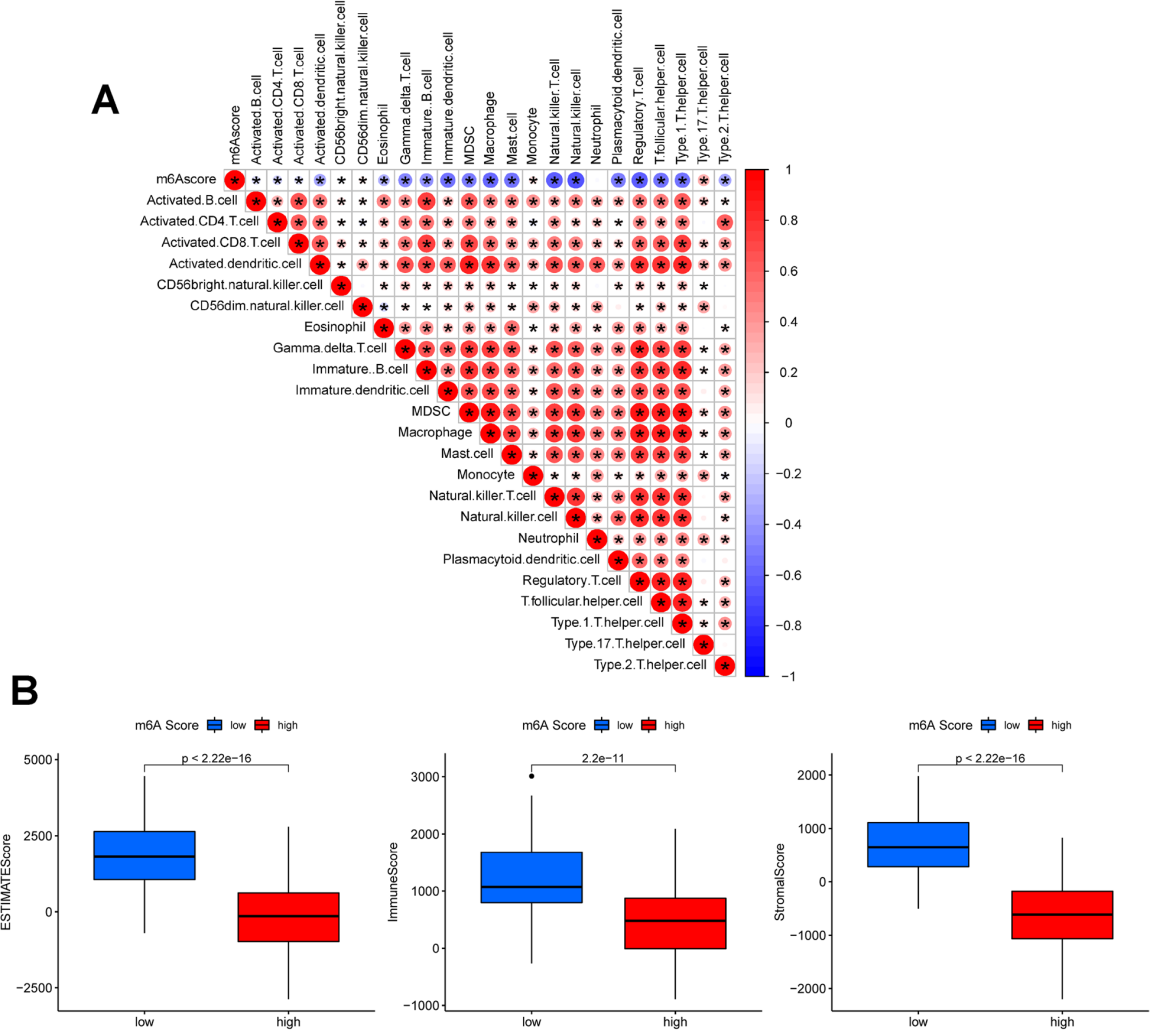
The correlationship of m6Ascore with TME. **A** The correlations between m6Ascore and immune cells analyzed by Spearman analysis. The negative correlation and positive correlation were marked with blue and red star, respectively. **B** The immune score, stromal score and ESTIMATE score of COAD patients were higher in the low- m6Ascore group than that of high-m6Ascore group (*P* < 0.05)

As shown in Fig. 5**A**, m6Ascore was negatively correlated with infiltration of most immune cells except for CD56 bright natural killer cells, monocyte and type 17 T helper cells. Our results indicated that patients with low-m6Ascore had higher immune score, stromal score and ESTIMATE score than those with high-m6Ascore (*P* < 0.05, Fig. 5B).

### The value of m6Ascore in predicting the response of patients to chemotherapy, targeted therapy and immunotherapy treatments

**Fig. 6:**
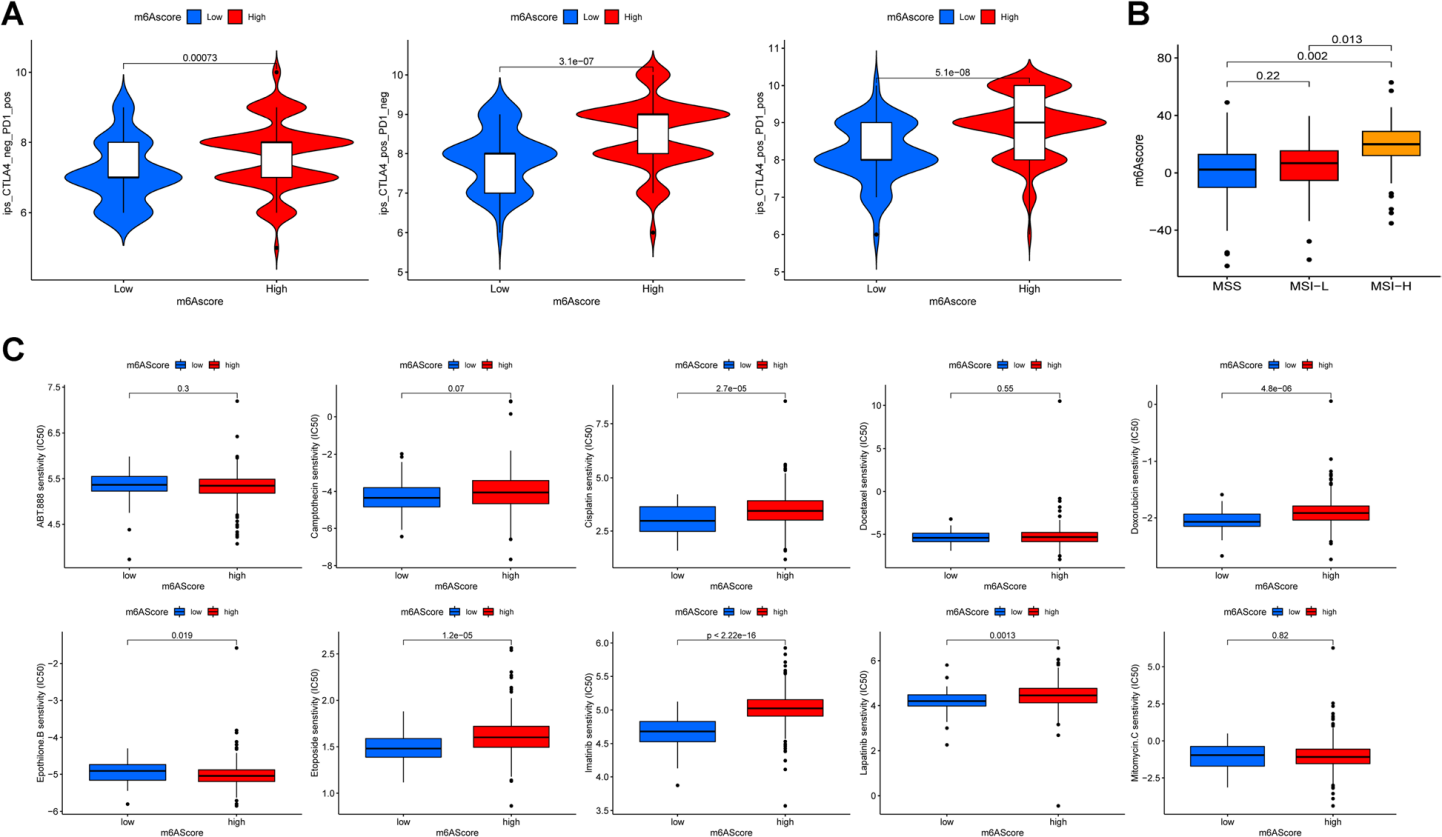
The value of m6Ascore in predicting response of patients to therapeutic treatments. **A** The IPS scores of anti-PD1 and anti-CTLA4 alone and in combination use were higher in high-m6Ascore group which demonstrated that patients in this group had great sensitivity to anti-PD1 and anti-CTLA4 treatments (*P* < 0.001). **B** The m6A score was higher in patients with MSI-H group who was know to be more sensitive to immunotherapy. **C** The pRRophetic algorithm was used to predict the sensitivity of 15 commonly used drugs in high- and low-m6Ascore group patients. The lower IC_50_ level indicates more sensitive to the drugs

Our results revealed that patients with a high-m6Ascore exhibited significant higher IPS score across all treatment groups (anti-PD1 alone, anti-CTLA4 alone and in combination) (*P* < 0.001, Fig. 6A). The results strongly suggested that the m6Ascore was associated with the response to immunotherapy and could potentially predict the prognosis of COAD patients treated with anti-PD-1 and anti-CTLA4. Moreover, we found that the patients in the MSI-H group had a higher m6Ascore, indicating greater sensitivity to anti-CTLA4 and anti-PD1 treatments (Fig. 6B).

The pRRophetic algorithm was used to forecast the sensitivity of 15 commonly used drugs in patients with high- and low-m6Ascores. A lower IC_50_ level indicates greater sensitivity to the drug. As shown in Fig. 6C, patients in the high-m6Ascore group were more responsive to epothilone.B and sorafenib (*P* < 0.05), while patients in the low-m6Ascore group were more sensitive to cisplatin, doxorubicin, etoposide, imatinib, lapatinib, OSI.906 and PHA.665752 (*P* < 0.05). There were no significant differences in the sensitivity to ABT.888, camptothecin, docetaxel, mitomycin.C, paclitaxel and sunitinib based on their m6Ascore (*P* > 0.05). These findings indicated that the m6Ascore might serve as a predictor of chemotherapy, targeted therapy and immunotherapy responses.

## Discussion

Numerous studies have shown that m6A regulators play a crucial role in various biological processes, including innate immunity, inflammation and anti-tumor effects by mediating m6A modifications [22–24]. The expression of these regulators is associated with the occurrence and progression of various cancers, such as gastric cancer, breast cancer, liver cancer, prostate cancer and so on, suggesting that they can be potential targets for tumor treatment [25–29]. Our study revealed that 23 m6A regulators were significantly dysregulated, indicating that these m6A regulators could also play critical roles in COAD tumorigenesis, consistent with previous findings. Yang et al. [30] have reported that ALKBH5 plays an important role in inhibiting the metastasis of colon cancer. The increased expression of METTL3, which mediates m6A modification on JAK1 mRNA, is shown to be correlated with a poor prognosis in colon cancer patients. KIAA1429 (also named VIRMA) was reported to be overexpressed in CRC by Zhou et al., and participate in the cell proliferation and migration in CRC cell lines by increasing the expression of SIRT1 mRNA in an m6A-dependent manner. In addition, some mutations of m6A regulators are commonly found in COAD patients, especially ZC3H13. ZC3H13 is a canonical CCCH zinc finger protein which plays a role in modulating RNA m6A methylation in the nucleus as an m6A writer. Somatic frameshift mutation of ZC3H13 have been found in 3.3% and 15.2% of gastric cancer and colorectal cancer with MSI-H. The mutations might contribute to the progression of these cancers by regulating cell cycle and DNA damage response [31, 32].

Several studies [33–35] have demonstrated that m6A regulators can affect the prognosis of CRC by constructing prognostic models based on lncRNAs and mRNAs related to m6A regulators. However, the prognostic value of m6A regulators themselves and their relationship with immune regulation and TME are still unclear. In this study, we identified 12 m6A regulators which were associated with survival of COAD patients. Among them, CBL1, IGF2BP1, LRPPRC, YTHDC2 and YTHDF1 were positively correlated with OS, while ALKBH5, HNRNPC, FTO, WTAP, HNRNPA2B1, METTL3 and ZC3H13 were negatively correlated. By stratifying the patients based on the expression of m6A regulators, we further investigated the the relationship between m6A regulators expression and the outcome of patients. We observed that the expression of m6A regulators except for ALKBH5, was significantly upregulated in cluster B patients who had worse clinical outcome than those in cluster A. To gain insight into the underlying biological molecular mechanism of these two clusters, we performed GSVA and ssGSEA analyses and found that in cluster B, several carcinogenic activation pathways, such as TGF-β signaling pathway, pathway in cancer, basal cell carcinoma were enriched, indicating that these pathways might contribute to the poor survival in this group. On the contrary, the cluster A subgroup exhibited enrichment of several anti-tumor cells, such as activated B cell, activated CD8 T cell, CD56 bright natural killer cell, natural killer cell and natural killer T cell, providing a new direction for understanding the role of m6A regulators in influencing immune infiltration and suppressing tumor progression.

To further explore the role of m6A regulators in survival, we first screened DEGs between the two m6A clusters and selected survival-associated DEGs. Next, we further calculated the m6Ascore for each patient based on these DEGs using PCA algorithm and divided the patients into high- and low-m6Ascore groups. Patients with a high-m6Ascore had a better survival time which was validated by an independent cohort. The AUC was 0.775 in training cohort and 0.701 in validation cohort. The m6Ascore was associated with survival status, clinical stage and TNM stages of patients and was negatively correlated with anti-tumor immune cells. Additionally, patients in m6A cluster A had a higher m6Ascore. All the results were consistent and confirmed that m6A regulators could predict clinical outcome of COAD patients, indicating that it might serve as a potential prognostic marker. Xiong et al [21] systematically analyzed 15 m6A regulators in glioblastoma (GM) and created a GM-score by PCA based on prognosis-related m6A methylation pattern signature genes. The AUC of GM-score for predicting 1-, 2-, and 3-year survival was 0.650, 0.617 and 0.710, respectively. The GM-score could identify m6A modification patterns in individual patients, resulting in a more personalization and efficacious anti-tumor immunotherapy strategy. Comparing with this research, our study employed the same algorithm but based on 27 m6A regulators and constructed a valuable prognostic model for COAD patients which was also validated by an independent cohort.

Previous studies have reported that m6A regulators played an important role in modulating immune cell infiltration in TME and consequently promote immune escape [36, 37]. Therefore, exploring the relationship between m6A regulators and the TME in COAD patients holds great promise. Our study showed that the immune score, stromal score and ESTIMATE score were higher in low-m6Ascore group. The patients with high-m6Ascore exhibited significant higher IPS scores in anti-PD1, anti-CTLA4 treatment alone and in combination, implying a favorable response to these treatments. It is also well known that microsatellite stability (MSS) and microsatellite instability (MSI) are important factors that affect the effectiveness of immunotherapy in solid tumors. Several previous studies have shown that patients with MSI are extremely sensitive to immunotherapy. Our results demonstrated that the patients in the MSI-H group had a higher m6Ascore, who showed great sensitivity to anti-CTLA and anti-PD1 treatments.

Systemic therapies, including chemotherapy, targeted therapy and immunotherapy, are widely used to treat patients with advanced and metastatic CRC. However, the common chemotherapy drugs used in CRC patients have a relatively low pathological complete response rate of about 10–20%, highlighting the need for more effective treatments. Targeted therapy and immunotherapy have emerged as essential treatment options for COAD. In our experiments, we found that pothilone.B and sorafenib were more effective in patients with a high m6Ascore, while cisplatin, etoposide, imatinib, lapatinib, OSI.906 and PHA.665752 were more effective in patients with a low-m6Ascore. These findings could offer valuable insights into the investigation of tumor sensitivity to diverse drugs and thereby aid in providing personalized clinical drug therapy for COAD patients.

## Conclusion

In this study, we conducted a comprehensive analysis of 27 m6A regulators in COAD, including expression level, CNV changes, mutations and prognostic values. We also developed a method to calculate the m6A score of each patient and established its potential for predicting patient prognosis, which was related to the TME and could provide new strategies for personalized immunotherapy and prognosis prediction. These findings offered promising avenues for large-scale, prospective, multicenter studies in patients samples and provided informative directions for mechanistic studies on the TME and immune cell infiltration in COAD.

### Supplementary Information

**Additional file 1:** **Supplementary Figure 1.** Overview of the study.

**Additional file 2:** **Supplementary Figure 2.** The expression level, CNV changes and mutations of m6A regulators in COAD. (A) The difference of mRNA expression level of m6A regulators between COAD tumor and non-tumor tissues in TCGA cohort. The results showed that 23 out of 27 regulators were dysregulated in COAD. (B) The location on different chromosomes and CNV changes of m6A regulators. The outer layer represented the chromosomes and the inner layer was the location of m6A regulators. Red dots represented more CNV gain and blue dots represented more loss frequency. (C) The gain and loss of CNV frequency of m6A regulators were represented by red and green dots, respectively.

**Additional file 3:** **Supplementary Figure 3.** The genetic alterations of m6A regulators. (A) A total of 130 out of 399 (32.58%) COAD patients occurred genetic mutation. The number on the right demonstrated the mutation frequency of each m6A regulator. (B) The differential expression level of ALKBH5, METTL3, YTHDF1 and ZNF217 between ZC3H13 wild and mutation groups (*P* < 0.05).

**Additional file 4:** **Supplementary Figure 4. **K-M survival curves of survival associated m6A regulators for COAD patients. The patients were classified into "high" and "low" group according to the median level of each m6A regulator.

**Additional file 5:** **Supplementary Figure 5.** The correlation of the m6A regulators and their prognosis prediction roles in COAD. The left circle displayed three RNA modification types (eraser, reader and writer) of m6A regulators in different colors. The purple color and green color in the right circle indicated high risk factor and low risk factor, respectively. The size of circle represented the prognosis effect of m6A regulators. The pink and blue lines connecting m6A regulators represented positive and negative correlation, respectively (*P* < 0.0001).

**Additional file 6:** **Supplementary Figure 6.** The results of GO enrichment analysis (A) and KEGG pathway analysis (B) of the two m6A clusters.

**Additional file 7:** **Supplementary Figure 7.** Representative results of GO enrichment analysis and KEGG pathway analysis of DEGs. (A) GO enrichment analysis. (B) KEGG pathway analysis.

**Additional file 8:** **Supplementary Table 1**. The list of survival-associated DEGs.

## Data Availability

All R scripts and data generated or analysed during this study are available from supplementary files or the corresponding author on reasonable request. Publicly available datasets were analyzed in this study. This data can be downloaded freely here: https://portal.gdc.cancer.gov/, https://www.ncbi.nlm.nih.gov/geo/query/acc.cgi?acc=GSE40967, https://www.ncbi.nlm.nih.gov/geo/query/acc.cgi?acc = GSE38832.

## References

[1] Sung H, Ferlay J, Siegel RL, Laversanne M, Soerjomataram I, Jemal A, Bray F (2021). Global Cancer Statistics 2020: GLOBOCAN Estimates of Incidence and Mortality Worldwide for 36 Cancers in 185 Countries. CA Cancer J Clin.

[2] Mutch MG (2007). Molecular profiling and risk stratification of adenocarcinoma of the colon. J Surg Oncol.

[3] Batista PJ, Molinie B, Wang J, Qu K, Zhang J, Li L, Bouley DM, Lujan E, Haddad B, Daneshvar K, Carter AC, Flynn RA, Zhou C, Lim KS, Dedon P, Wernig M, Mullen AC, Xing Y, Giallourakis CC, Chang HY (2014). m(6)A RNA modification controls cell fate transition in mammalian embryonic stem cells. Cell Stem Cell.

[4] Wang X, Lu Z, Gomez A, Hon GC, Yue Y, Han D, Fu Y, Parisien M, Dai Q, Jia G, Ren B, Pan T, He C (2014). N6-methyladenosine-dependent regulation of messenger RNA stability. Nature.

[5] Niu Y, Zhao X, Wu YS, Li MM, Wang XJ, Yang YG (2013). N6-methyl-adenosine (m6A) in RNA: an old modification with a novel epigenetic function. Genomics Proteomics Bioinformatics.

[6] Sun T, Wu R, Ming L (2019). The role of m6A RNA methylation in cancer. Biomed pharmacother..

[7] Liu X, Wang P, Teng X, Zhang Z, Song S (2021). Comprehensive Analysis of Expression Regulation for RNA m6A Regulators With Clinical Significance in Human Cancers. Front Oncol.

[8] Liu T, Yang S, Cheng YP, Kong XL, Du DD, Wang X, Bai YF, Yin LH, Pu YP, Liang GY (2020). The N6-Methyladenosine (m6A) Methylation Gene YTHDF1 Reveals a Potential Diagnostic Role for Gastric Cancer. Cancer Manag Res.

[9] Liu J, Sun G, Pan S, Qin M, Ouyang R, Li Z, Huang J (2020). The Cancer Genome Atlas (TCGA) based m(6)A methylation-related genes predict prognosis in hepatocellular carcinoma. Bioengineered.

[10] Zhang H, Hu J, Liu A, Qu H, Jiang F, Wang C, Mo S, Sun P (2021). An N6-Methyladenosine-Related Gene Set Variation Score as a Prognostic Tool for Lung Adenocarcinoma. Front Cell Dev Biol.

[11] Geng Y, Guan R, Hong W, Huang B, Liu P, Guo X, Hu S, Yu M, Hou B (2020). Identification of m6A-related genes and m6A RNA methylation regulators in pancreatic cancer and their association with survival. Ann Transl Med.

[12] Xu N, Chen J, He G, Gao L, Zhang D (2020). Prognostic values of m6A RNA methylation regulators in differentiated Thyroid Carcinoma. J Cancer.

[13] Mahajan UM, Langhoff E, Goni E, Costello E, Greenhalf W, Halloran C, Ormanns S, Kruger S, Boeck S, Ribback S, Beyer G, Dombroswki F, Weiss FU, Neoptolemos JP, Werner J, D'Haese JG, Bazhin A, Peterhansl J, Pichlmeier S, Büchler MW, Kleeff J, Ganeh P, Sendler M, Palmer DH, Kohlmann T, Rad R, Regel I, Lerch MM, Mayerle J (2018). Immune Cell and Stromal Signature Associated With Progression-Free Survival of Patients With Resected Pancreatic Ductal Adenocarcinoma. Gastroenterology.

[14] Sun Q, Zhang B, Hu Q, Qin Y, Xu W, Liu W, Yu X, Xu J (2018). The impact of cancer-associated fibroblasts on major hallmarks of pancreatic cancer. Theranostics.

[15] Fridman WH, Zitvogel L, Sautès-Fridman C, Kroemer G (2017). The immune contexture in cancer prognosis and treatment. Nat Rev Clin Oncol.

[16] Lin S, Xu H, Zhang A, Ni Y, Xu Y, Meng T, Wang M, Lou M (2020). Prognosis Analysis and Validation of m(6)A Signature and Tumor Immune Microenvironment in Glioma. Front Oncol.

[17] Chong W, Shang L, Liu J, Fang Z, Du F, Wu H, Liu Y, Wang Z, Chen Y, Jia S, Chen L, Li L, Chen H (2021). m(6)A regulator-based methylation modification patterns characterized by distinct tumor microenvironment immune profiles in colon cancer. Theranostics.

[18] Yao Y, Luo L, Xiang G, Xiong J, Ke N, Tan C, Chen Y, Liu X (2022). The expression of m(6)A regulators correlated with the immune microenvironment plays an important role in the prognosis of pancreatic ductal adenocarcinoma. Gland Surg.

[19] Ye W, Huang T (2022). Correlation analysis of m6A-modified regulators with immune microenvironment infiltrating cells in lung adenocarcinoma. PLoS ONE.

[20] Zhu W, Zhao L, Kong B, Liu Y, Zou X, Han T, Shi Y (2022). The methylation modification of m6A regulators contributes to the prognosis of ovarian cancer. Ann Transl Med.

[21] Xiong W, Li C, Wan B, Zheng Z, Zhang Y, Wang S, Fan J (2022). N6-Methyladenosine Regulator-Mediated Immue Patterns and Tumor Microenvironment Infiltration Characterization in Glioblastoma. Front Immunol.

[22] Chen XY, Zhang J, Zhu JS (2019). The role of m(6)A RNA methylation in human cancer. Mol Cancer.

[23] Shulman Z, Stern-Ginossar N (2020). The RNA modification N(6)-methyladenosine as a novel regulator of the immune system. Nat Immunol.

[24] Jian D, Wang Y, Jian L, Tang H, Rao L, Chen K, Jia Z, Zhang W, Liu Y, Chen X, Shen X, Gao C, Wang S, Li M (2020). METTL14 aggravates endothelial inflammation and atherosclerosis by increasing FOXO1 N6-methyladeosine modifications. Theranostics.

[25] Zhang C, Zhang M, Ge S, Huang W, Lin X, Gao J, Gong J, Shen L (2019). Reduced m6A modification predicts malignant phenotypes and augmented Wnt/PI3K-Akt signaling in gastric cancer. Cancer Med.

[26] Anita R, Paramasivam A, Priyadharsini JV, Chitra S (2020). The m6A readers YTHDF1 and YTHDF3 aberrations associated with metastasis and predict poor prognosis in breast cancer patients. Am J Cancer Res.

[27] Zhang C, Huang S, Zhuang H, Ruan S, Zhou Z, Huang K, Ji F, Ma Z, Hou B, He X (2020). YTHDF2 promotes the liver cancer stem cell phenotype and cancer metastasis by regulating OCT4 expression via m6A RNA methylation. Oncogene.

[28] Chen Y, Pan C, Wang X, Xu D, Ma Y, Hu J, Chen P, Xiang Z, Rao Q, Han X (2021). Silencing of METTL3 effectively hinders invasion and metastasis of prostate cancer cells. Theranostics.

[29] Li J, Xie H, Ying Y, Chen H, Yan H, He L, Xu M, Xu X, Liang Z, Liu B, Wang X, Zheng X, Xie L (2020). YTHDF2 mediates the mRNA degradation of the tumor suppressors to induce AKT phosphorylation in N6-methyladenosine-dependent way in prostate cancer. Mol Cancer.

[30] Yang P, Wang Q, Liu A, Zhu J, Feng J (2020). ALKBH5 Holds Prognostic Values and Inhibits the Metastasis of Colon Cancer. Pathol Oncol Res.

[31] Kim YR, Chung NG, Kang MR, Yoo NJ, Lee SH (2010). Novel somatic frameshift mutations of genes related to cell cycle and DNA damage response in gastric and colorectal cancers with microsatellite instability. Tumori.

[32] Zhu D, Zhou J, Zhao J, Jiang G, Zhang X, Zhang Y, Dong M (2019). ZC3H13 suppresses colorectal cancer proliferation and invasion via inactivating Ras-ERK signaling. J Cell Physiol.

[33] Li Z, Liu Y, Yi H, Cai T, Wei Y (2022). Identification of N6-methylandenosine related lncRNA signatures for predicting the prognosis and therapy response in colorectal cancer patients. Front Genet.

[34] Li W, Gao Y, Jin X, Wang H, Lan T, Wei M, Yan W, Wang G, Li Z, Zhao Z, Jiang X (2022). Comprehensive analysis of N6-methylandenosine regulators and m6A-related RNAs as prognosis factors in colorectal cancer. Mol Ther Nucleic Acids.

[35] Zeng H, Xu Y, Xu S, Jin L, Shen Y, Rajan KC, Bhandari A, Xia E (2021). Construction and Analysis of a Colorectal Cancer Prognostic Model Based on N6-Methyladenosine-Related lncRNAs. Front Cell Dev Biol.

[36] Yan G, An Y, Xu B, Wang N, Sun X, Sun M (2021). Potential Impact of ALKBH5 and YTHDF1 on Tumor Immunity in Colon Adenocarcinoma. Front Oncol.

[37] Zhou Y, Zhou H, Shi J, Guan A, Zhu Y, Hou Z, Li R (2021). Decreased m6A Modification of CD34/CD276(B7–H3) Leads to Immune Escape in Colon Cancer. Front Cell Dev Biol.

