# Supplementary material for: Comprehensive analysis of m6A regulators and relationship with tumor microenvironment, immunotherapy strategies in colorectal adenocarcinoma
Source: BMC Genom Data. 2023 Aug 11;24:44. doi: 10.1186/s12863-023-01149-y (PMC10422724; doi:10.1186/s12863-023-01149-y)
Supplement: Supplementary file 1 — Additional file 1: Supplementary Figure 1. Overview of the study. [file 12863_2023_1149_MOESM1_ESM.pptx]

## Slide 1
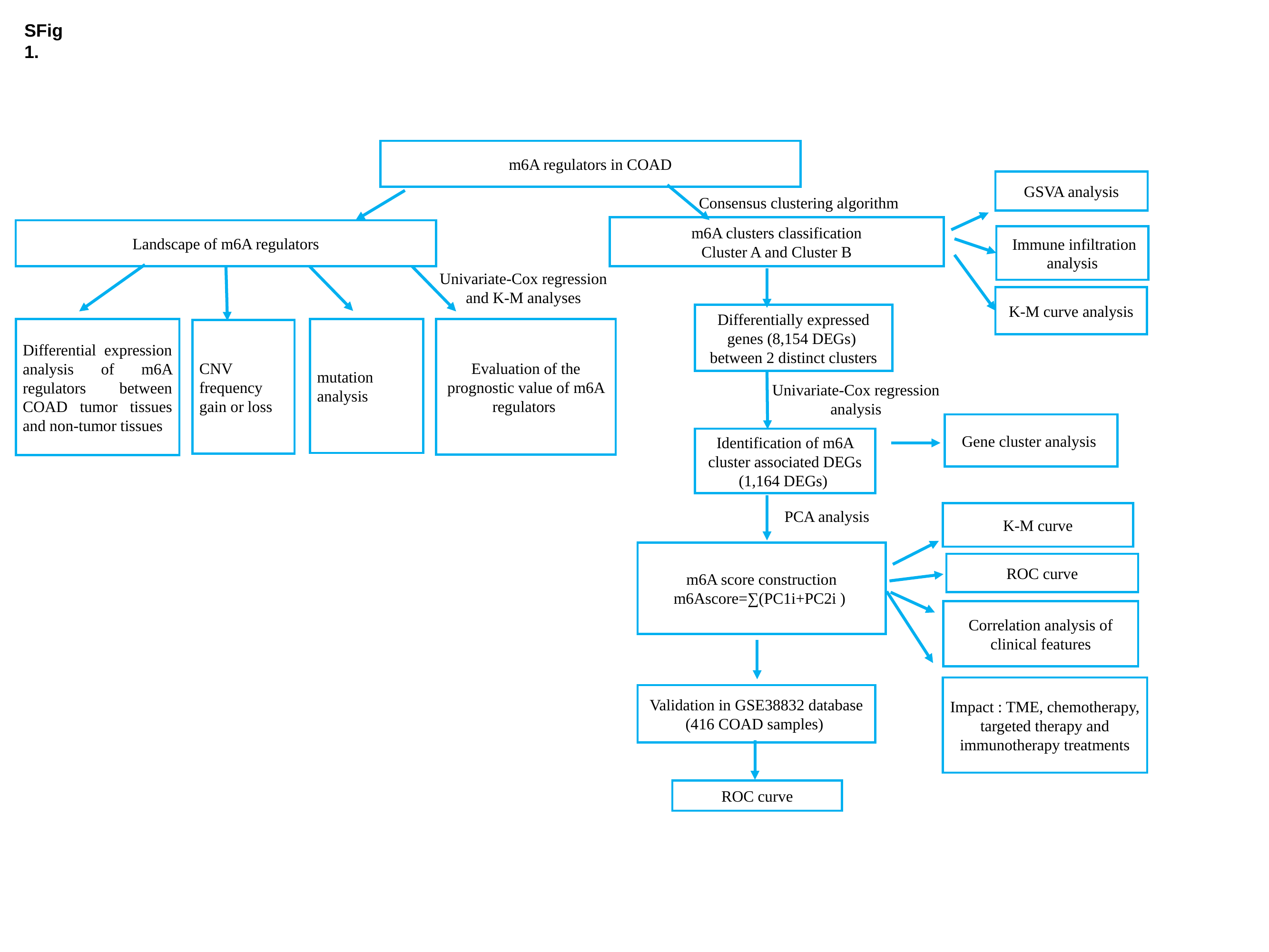

SFig 1.
m6A regulators in COAD
GSVA analysis
Consensus clustering algorithm
m6A clusters classification
Cluster A and Cluster B
Landscape of m6A regulators
 Immune infiltration analysis
Univariate-Cox regression and K-M analyses
K-M curve analysis
Differentially expressed genes (8,154 DEGs) between 2 distinct clusters
Differential expression analysis of m6A regulators between COAD tumor tissues and non-tumor tissues
mutation analysis
Evaluation of the prognostic value of m6A regulators
CNV frequency
gain or loss
Univariate-Cox regression analysis
Gene cluster analysis
Identification of m6A cluster associated DEGs (1,164 DEGs)
PCA analysis
K-M curve
m6A score construction
m6Ascore=∑(PC1i+PC2i )
ROC curve
Correlation analysis of clinical features
Impact : TME, chemotherapy, targeted therapy and immunotherapy treatments
Validation in GSE38832 database (416 COAD samples)
ROC curve
