# Supplementary figures and images for: Comprehensive analysis of m6A regulators and relationship with tumor microenvironment, immunotherapy strategies in colorectal adenocarcinoma
Source: BMC Genom Data. 2023 Aug 11;24:44. doi: 10.1186/s12863-023-01149-y (PMC10422724; doi:10.1186/s12863-023-01149-y)

## Slide 1
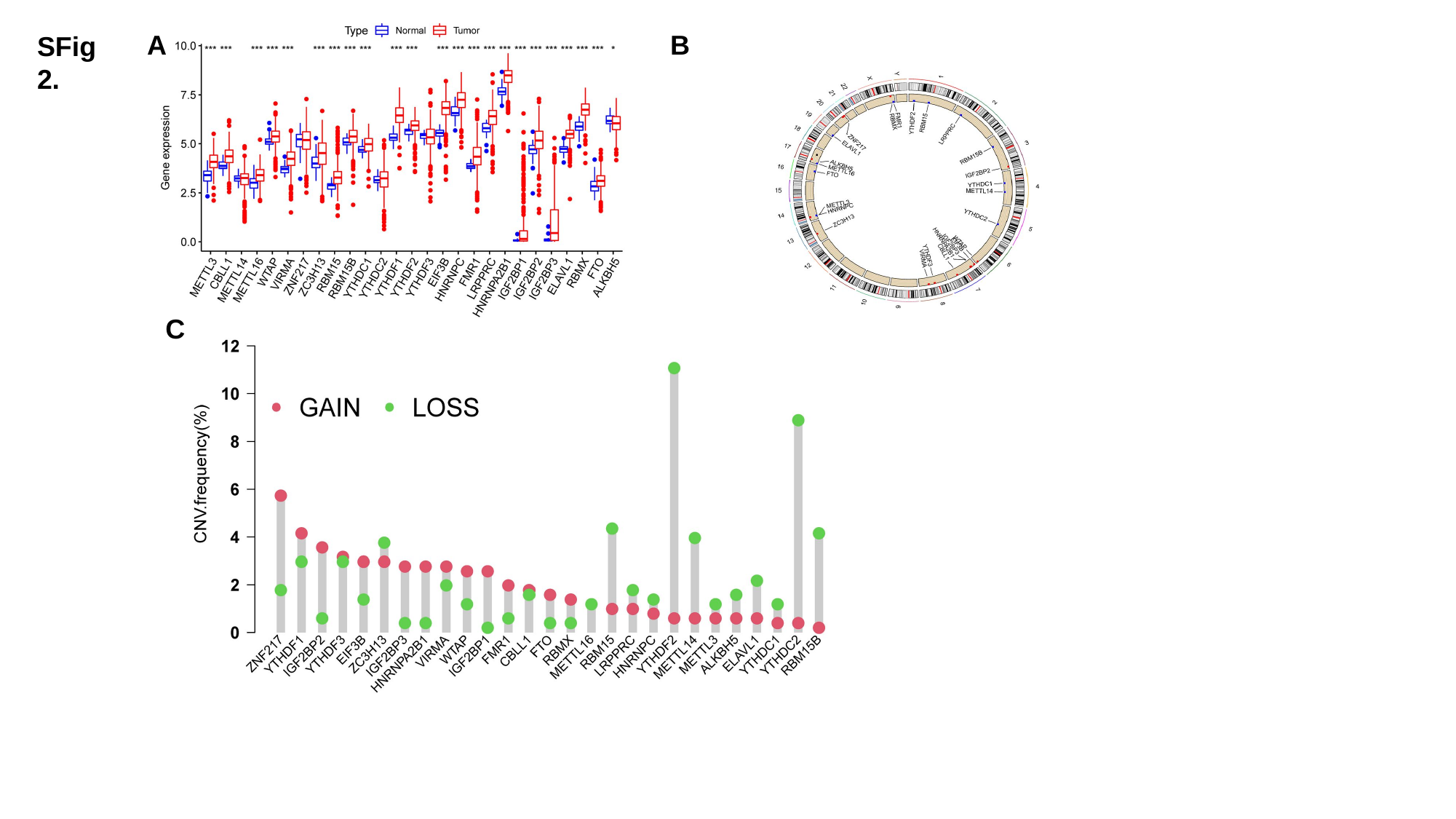

A
B
SFig 2.
C

Supplement: Supplementary file 2 — Additional file 2: Supplementary Figure 2. The expression level, CNV changes and mutations of m6A regulators in COAD. (A) The difference of mRNA expression level of m6A regulators between COAD tumor and non-tumor tissues in TCGA cohort. The results showed that 23 out of 27 regulators were dysregulated in COAD. (B) The location on different chromosomes and CNV changes of m6A regulators. The outer layer represented the chromosomes and the inner layer was the location of m6A regulators. Red dots represented more CNV gain and blue dots represented more loss frequency. (C) The gain and loss of CNV frequency of m6A regulators were represented by red and green dots, respectively. [file 12863_2023_1149_MOESM2_ESM.pptx]

## Slide 1
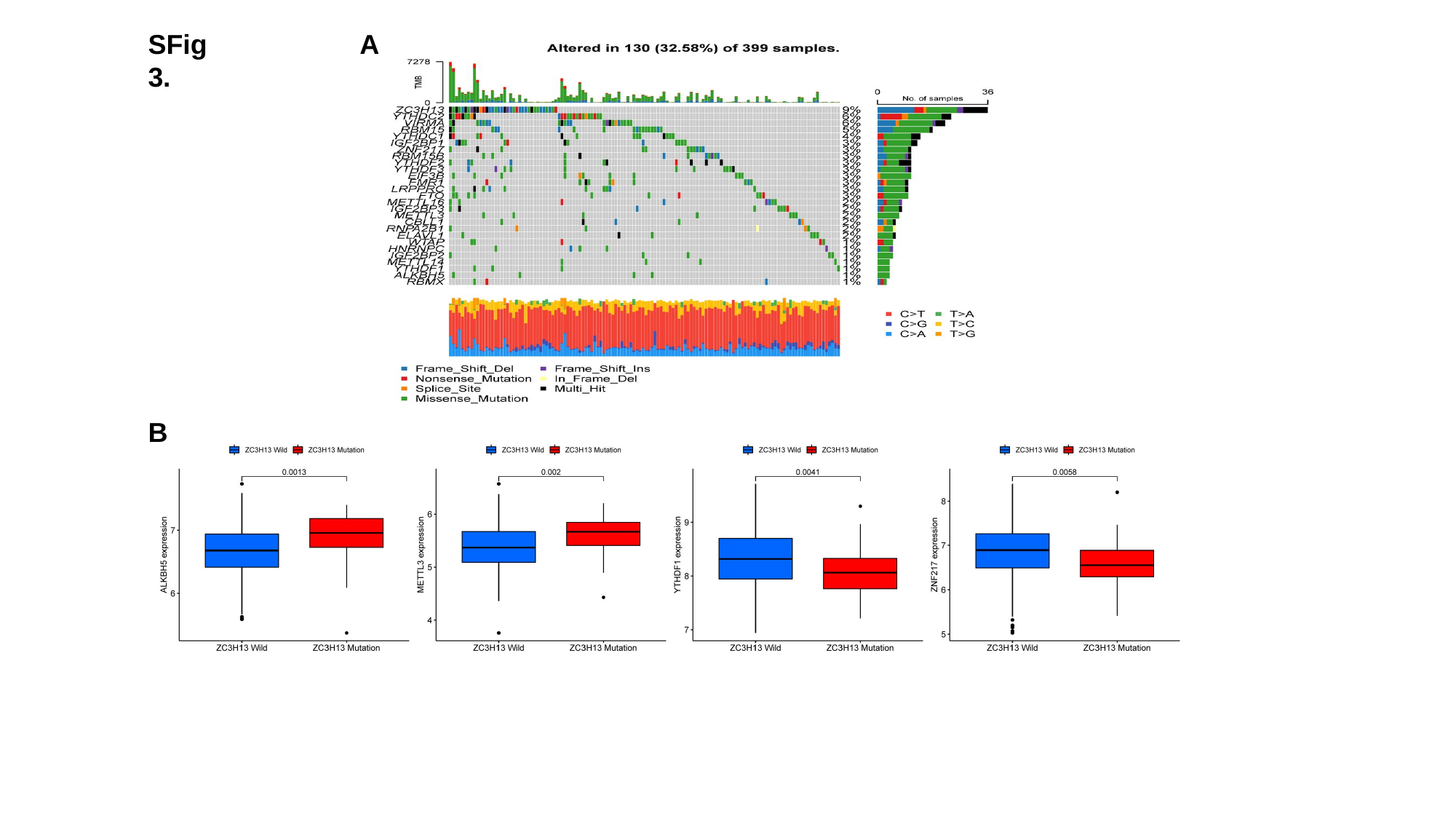

SFig 3.
A
B

Supplement: Supplementary file 3 — Additional file 3: Supplementary Figure 3. The genetic alterations of m6A regulators. (A) A total of 130 out of 399 (32.58%) COAD patients occurred genetic mutation. The number on the right demonstrated the mutation frequency of each m6A regulator. (B) The differential expression level of ALKBH5, METTL3, YTHDF1 and ZNF217 between ZC3H13 wild and mutation groups (P < 0.05). [file 12863_2023_1149_MOESM3_ESM.pptx]

## Slide 1
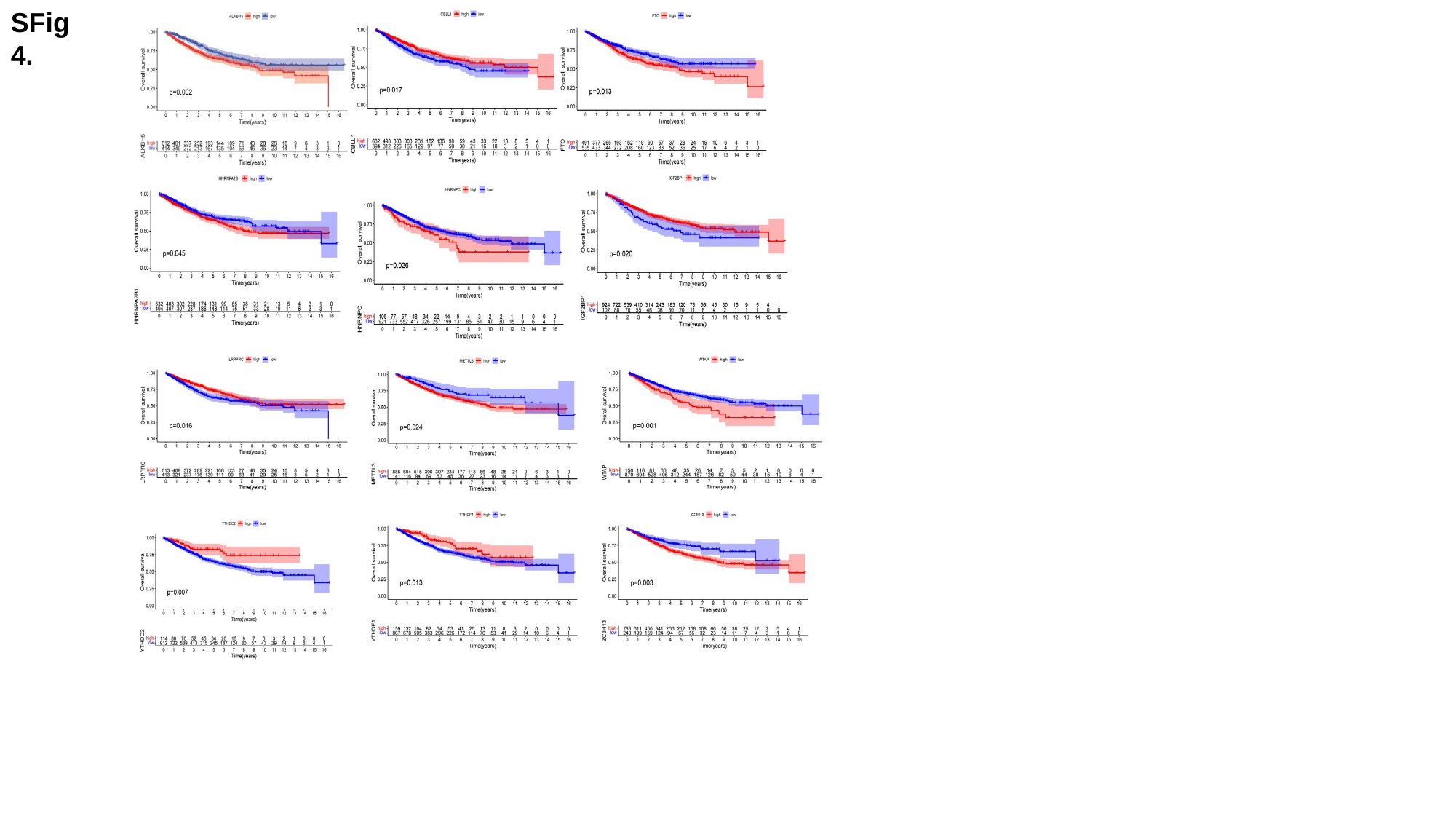

SFig 4.

Supplement: Supplementary file 4 — Additional file 4: Supplementary Figure 4. K-M survival curves of survival associated m6A regulators for COAD patients. The patients were classified into "high" and "low" group according to the median level of each m6A regulator. [file 12863_2023_1149_MOESM4_ESM.pptx]

## Slide 1
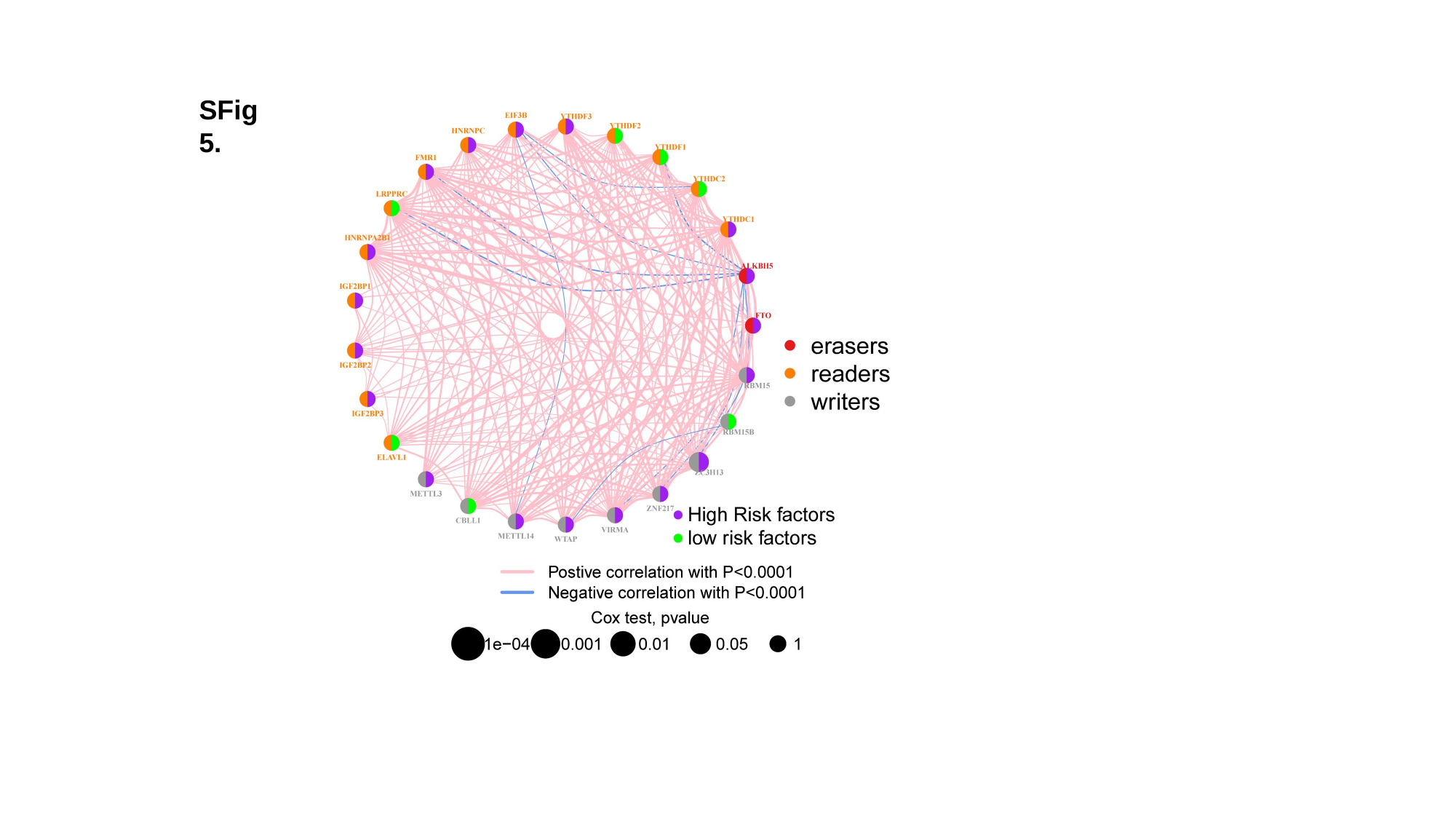

SFig 5.

Supplement: Supplementary file 5 — Additional file 5: Supplementary Figure 5. The correlation of the m6A regulators and their prognosis prediction roles in COAD. The left circle displayed three RNA modification types (eraser, reader and writer) of m6A regulators in different colors. The purple color and green color in the right circle indicated high risk factor and low risk factor, respectively. The size of circle represented the prognosis effect of m6A regulators. The pink and blue lines connecting m6A regulators represented positive and negative correlation, respectively (P < 0.0001). [file 12863_2023_1149_MOESM5_ESM.pptx]

## Slide 1
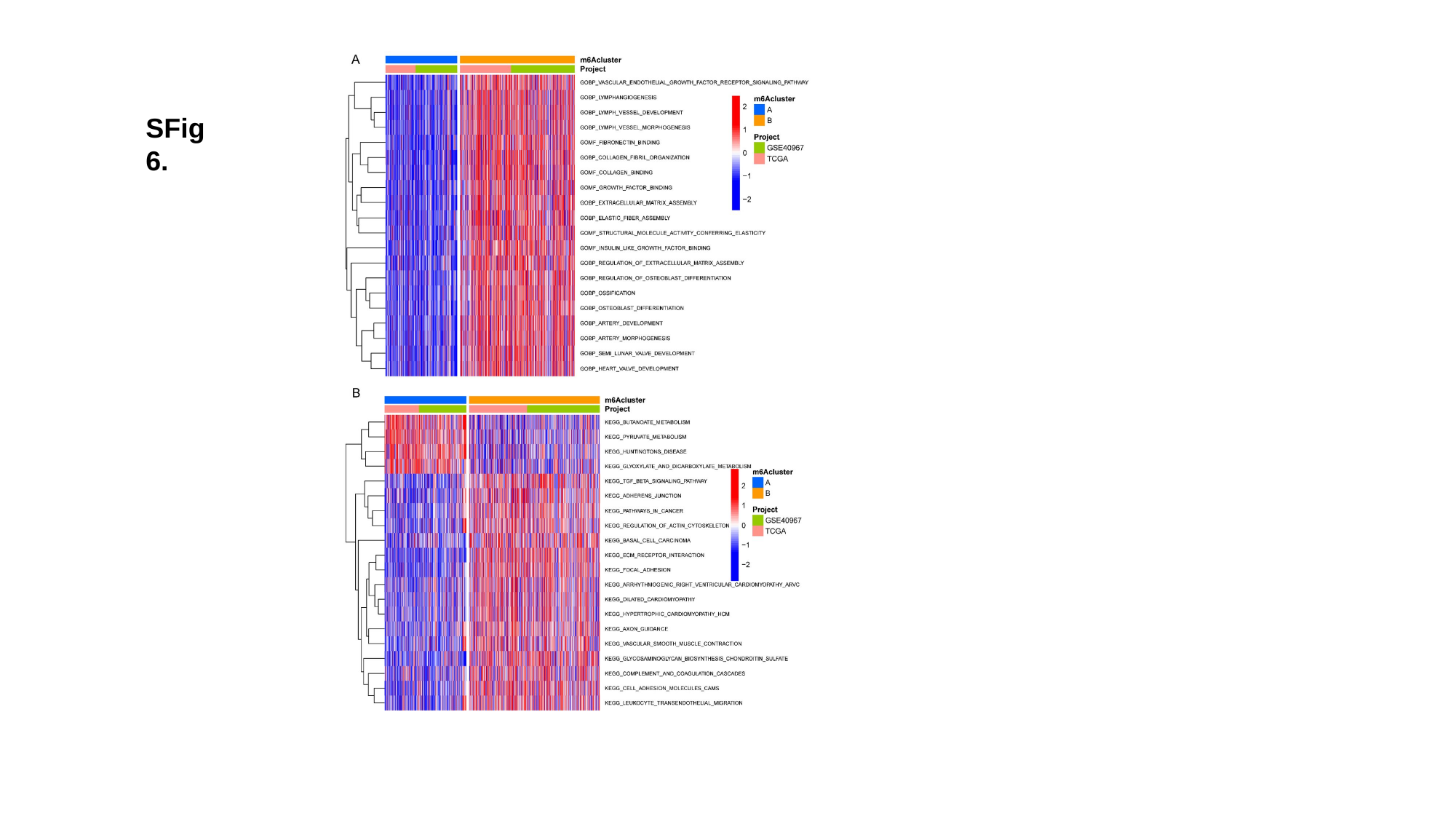

SFig 6.

Supplement: Supplementary file 6 — Additional file 6: Supplementary Figure 6. The results of GO enrichment analysis (A) and KEGG pathway analysis (B) of the two m6A clusters. [file 12863_2023_1149_MOESM6_ESM.pptx]

## Slide 1
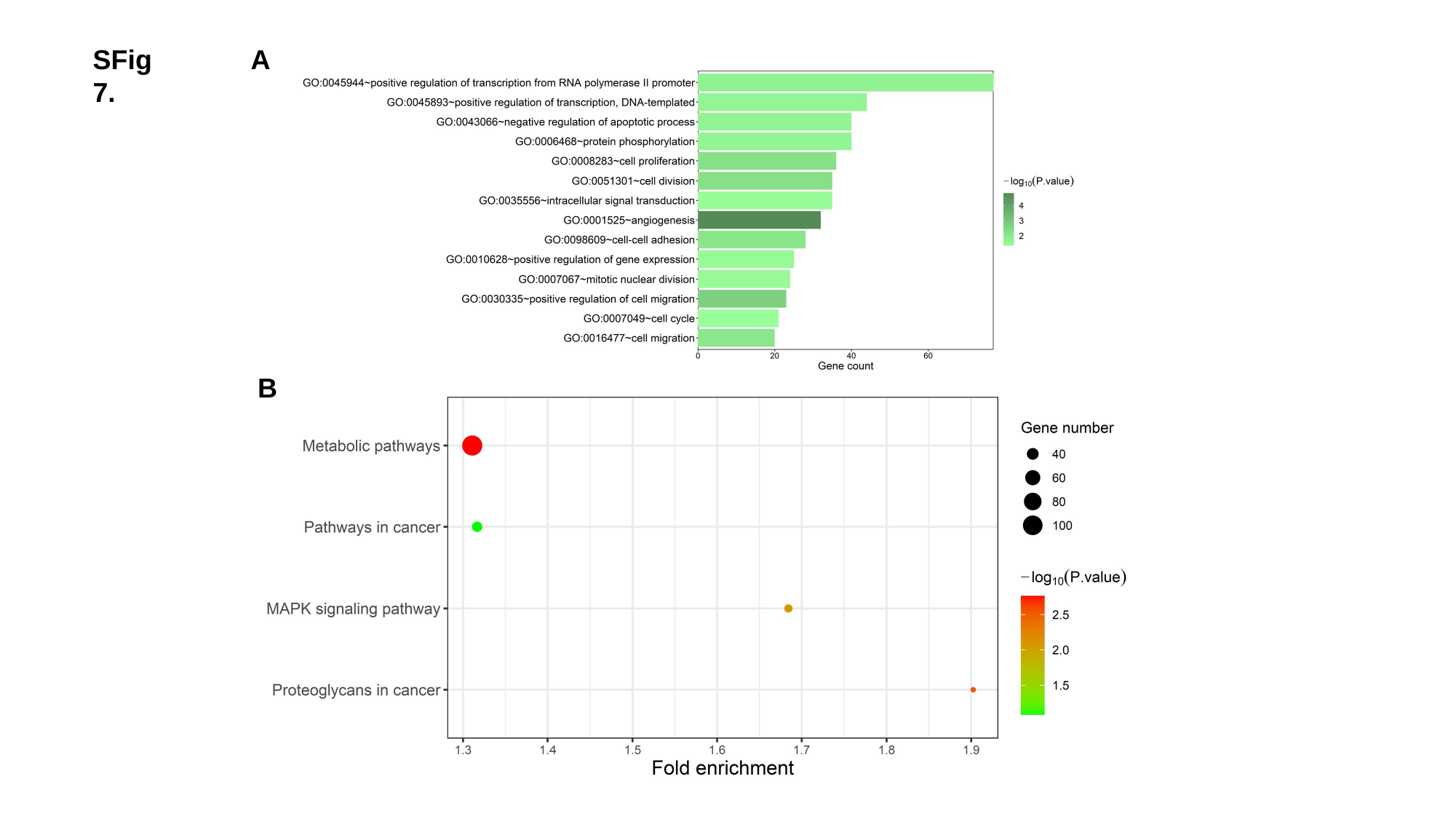

SFig 7.
A
B

Supplement: Supplementary file 7 — Additional file 7: Supplementary Figure 7. Representative results of GO enrichment analysis and KEGG pathway analysis of DEGs. (A) GO enrichment analysis. (B) KEGG pathway analysis. [file 12863_2023_1149_MOESM7_ESM.pptx]
