# Supplementary material for: Comprehensive analysis of m6A regulators and relationship with tumor microenvironment, immunotherapy strategies in colorectal adenocarcinoma
Source: BMC Genom Data. 2023 Aug 11;24:44. doi: 10.1186/s12863-023-01149-y (PMC10422724; doi:10.1186/s12863-023-01149-y)
Supplement: Supplementary file 8 — Additional file 8: Supplementary Table 1. The list of survival-associated DEGs. [file 12863_2023_1149_MOESM8_ESM.pdf]

Supplementary Table 1 The list of survival-associated DEGs

Gene	HR	HR.95L	HR.95H	P value
RBL1	0.803246	0.65513124	0.984847	0.03513591
ZC3H13	1.2735181	1.04006383	1.5593739	0.01927514
LBR	0.8325525	0.70047741	0.9895304	0.03758192
KBTBD2	1.3405034	1.02805198	1.7479168	0.03044213
ZNF12	1.2332632	1.02121726	1.4893383	0.02940069
GLS	1.3172333	1.07923576	1.6077151	0.00672975
DSN1	0.8232992	0.68569309	0.9885203	0.03718747
QSER1	1.2261055	1.01166438	1.4860014	0.0376914
EPB41L5	0.7771828	0.60858047	0.9924951	0.04334812
RBBP6	1.311112	1.02492261	1.6772142	0.0310922
OSBPL3	1.3047688	1.08922595	1.5629646	0.00388055
AHCTF1	1.2244346	1.00088482	1.4979148	0.04900333
MAP3K7	1.3276217	1.02091045	1.7264779	0.03448383
SLC25A36	1.2574984	1.03027712	1.5348318	0.02424005
MOSPD2	1.2942944	1.04458454	1.6036978	0.01833343
TAF1	1.3162663	1.04058875	1.6649775	0.02191846
DDX52	0.749931	0.59138342	0.9509847	0.01756405
PTPN12	1.4427021	1.15640984	1.7998718	0.00116382
CCDC82	1.3151214	1.03248877	1.6751215	0.02648988
AURKA	0.7856848	0.65573124	0.9413927	0.00893167
E2F3	1.3144528	1.03457277	1.6700479	0.02520862
PIK3CA	1.4109684	1.11338771	1.788085	0.00438979
YAP1	1.2864557	1.05226174	1.5727725	0.01401699
ASAP1	1.2101536	1.0200843	1.435638	0.02866461
PLAGL2	0.8353307	0.73441819	0.950109	0.00616148
CWF19L2	1.4015423	1.07073458	1.8345543	0.01399037
CNOT1	0.7550221	0.58603772	0.9727332	0.02971842
BRIP1	0.8363783	0.70314988	0.9948499	0.04356111
MSANTD2	1.2714866	1.03892861	1.5561013	0.01977871
RAD18	0.7805689	0.61254561	0.9946816	0.04516856
RC3H2	1.3334137	1.0275662	1.7302944	0.0304243
KIF15	0.8064082	0.69980853	0.9292459	0.00293591
KIDINS220	1.4430257	1.11094329	1.874374	0.00598834
MORF4L2	1.2382019	1.0080695	1.5208713	0.04169291
SF3B1	1.3735082	1.03852551	1.8165416	0.02608237
FAM3C	1.4439976	1.16229702	1.7939726	0.00090578
MTR	1.3962882	1.108587	1.7586537	0.00457342
JRKL	1.3323719	1.05466149	1.6832082	0.01611842
TUBE1	1.2949651	1.00242924	1.6728708	0.0478683
SSX2IP	0.763283	0.63726934	0.9142147	0.00334402
CHORDC1	1.2318224	1.00512185	1.5096543	0.04451639
CLASP1	1.4011603	1.02135306	1.9222052	0.03653354
LRCH1	1.3469937	1.08993044	1.664686	0.00583354
TTC14	1.2360151	1.04032324	1.4685179	0.01597497
PLK4	0.8244083	0.68540291	0.991605	0.04041873
CCNL1	1.2434796	1.00425749	1.5396864	0.04561536
FAM122C	1.4782933	1.03383069	2.1138385	0.03216856
STARD7	0.6819126	0.47869782	0.9713953	0.03394514
ZNF322	1.3083968	1.0208203	1.6769868	0.03377981
RNF114	0.7427404	0.59068534	0.9339376	0.01093532
KDM3A	1.3203862	1.00535512	1.7341333	0.04567696
SAMD8	1.2579935	1.00626133	1.5727004	0.04393024

KIF2A	0.7934232	0.64913416	0.9697847	0.0238486
RNFT1	0.806264	0.67154782	0.9680051	0.02096752
CCDC34	0.789061	0.63537672	0.9799184	0.03207252
MYC	0.8721613	0.76268449	0.9973525	0.0456406
SERINC5	0.7902032	0.64333379	0.970602	0.02480814
RMI1	0.823074	0.68671045	0.986516	0.03512966
LIG4	1.2147389	1.01423527	1.45488	0.03455473
TUG1	1.230466	1.00029877	1.5135944	0.04967045
PSMD12	0.7705219	0.59971109	0.9899833	0.04148055
YEATS2	1.4950168	1.11716895	2.00066	0.00682331
HOOK1	0.7700357	0.6620442	0.8956427	0.00070011
COPS8	1.3970949	1.03470134	1.886413	0.02906374
PLAG1	1.1815317	1.01289654	1.3782426	0.03374949
PICALM	1.5078228	1.16627633	1.9493918	0.00172619
FASTKD3	0.7947228	0.64288822	0.9824171	0.03367442
GIGYF2	1.3697118	1.00668944	1.8636436	0.04524175
ZNF449	1.3261219	1.00966283	1.7417689	0.04244868
PARPBP	0.7641602	0.64179968	0.909849	0.00251852
POP1	0.7667397	0.63279437	0.9290376	0.00670122
KMT2A	1.4452415	1.12373791	1.8587278	0.00412172
ABCC4	0.8056552	0.68535291	0.9470745	0.00881922
RAB3IP	0.7487878	0.56609321	0.9904432	0.04263635
CBL	1.4934185	1.13519958	1.9646755	0.00415449
HSPBAP1	1.3043414	1.0423677	1.6321558	0.0201949
CHCHD3	0.7149198	0.51315448	0.9960165	0.04730532
DHX15	0.6993589	0.5295076	0.9236937	0.01176424
FXR1	1.5905658	1.20863058	2.0931951	0.00092489
KPNA5	1.2244539	1.00688121	1.4890409	0.04249229
RSRC2	1.3514072	1.0434683	1.7502222	0.02246212
ZNF117	1.3301217	1.13527749	1.5584064	0.00041565
FCGBP	0.9507457	0.90736365	0.996202	0.03403501
CAMSAP2	1.4151552	1.15155438	1.7390964	0.00096087
UBQLN2	1.3022802	1.02212999	1.6592152	0.03259197
ZNF736	1.2250993	1.02815184	1.4597731	0.02318074
MUC2	0.952509	0.90962051	0.9974197	0.03846422
CDK5RAP1	0.7291957	0.57236555	0.9289979	0.01058702
ATP6AP2	1.3760522	1.05998144	1.7863706	0.01650944
ZSCAN29	1.3911148	1.08892588	1.7771644	0.00824848
TMEM61	0.8725059	0.7653129	0.9947127	0.04142724
KIAA2026	1.385396	1.07511675	1.7852219	0.01174103
UQCRC1	0.6207467	0.48696436	0.7912828	0.00011801
C4orf46	0.8238453	0.69497741	0.9766089	0.02556986
PNISR	1.2078488	1.00472334	1.4520402	0.04441814
DEPDC1B	0.8479101	0.73321163	0.9805512	0.02609318
MAP4K5	1.2405378	1.00858755	1.5258309	0.04125781
ZNF514	1.1883163	1.01997148	1.3844462	0.02685113
CDC42EP5	0.8481382	0.76089558	0.9453838	0.00293866
CARF	1.3816135	1.03472981	1.8447867	0.02842211
AGGF1	0.752603	0.57693121	0.9817657	0.03611291
RANBP9	1.4950893	1.08616607	2.0579652	0.01362682
MAPKAPK5	0.6685592	0.50275637	0.8890417	0.00562754
PIGU	0.7896378	0.65511718	0.9517807	0.0131895
RANBP6	1.237061	1.00998892	1.5151848	0.03978024
ZNF180	0.6543752	0.49692157	0.8617192	0.00253018

FAM173B	0.7442128	0.55969374	0.9895638	0.04214079
ANG	0.8577087	0.75620967	0.9728311	0.01691203
TEAD1	1.2494226	1.0081967	1.5483654	0.04189632
AKR7A3	0.8076596	0.69751413	0.9351984	0.00429605
WAC	1.4150908	1.03093151	1.9424004	0.03167637
PRIM1	0.782458	0.66000294	0.9276329	0.0047276
HERPUD2	1.3374942	1.02773873	1.7406084	0.03050052
BMPR2	1.2902263	1.05187155	1.5825924	0.01447531
ETHE1	0.7883644	0.66782576	0.9306595	0.00497297
CNOT6L	0.770911	0.60155779	0.9879412	0.03979935
SPIN1	1.357065	1.05508763	1.7454715	0.01742917
RUFY3	1.3218065	1.01107024	1.7280425	0.04130233
PDP2	0.7604623	0.5932883	0.9747418	0.0306224
NFS1	0.7251702	0.57983978	0.906926	0.00486113
ZNF738	1.2205406	1.01538538	1.4671466	0.03378921
BICD1	1.465888	1.17707581	1.8255643	0.00063501
MRPL54	0.758918	0.60538489	0.951389	0.01675346
CEP350	1.2665652	1.00849106	1.5906807	0.04208339
AKAP8	0.6592153	0.44895032	0.9679575	0.03349249
GSTCD	0.7764084	0.60511295	0.9961943	0.04659692
TRIM23	1.3399191	1.05348706	1.7042291	0.01709794
KDELC2	1.2235328	1.01953842	1.4683435	0.03016658
CHN2	0.813146	0.71678233	0.9224648	0.00130904
ZNF160	1.3020927	1.00734578	1.6830817	0.04381506
ATP6V1H	1.3523693	1.03873776	1.7606973	0.02494295
ZNF823	0.7563398	0.61823604	0.9252935	0.00663261
KAT6A	1.289588	1.01734244	1.6346877	0.03554651
MAD2L1	0.8512146	0.73307721	0.9883902	0.03458799
OSTM1	1.2622675	1.01411579	1.5711415	0.03702639
PAK2	1.4719263	1.09774649	1.9736497	0.00979058
AMOTL2	1.2011917	1.00375281	1.4374668	0.04540869
PRDM4	0.7276236	0.53300224	0.9933093	0.04525933
PSMA7	0.7879198	0.65015266	0.9548797	0.01506516
SPINK4	0.9442937	0.90542329	0.9848328	0.00752686
TMEM125	0.8045427	0.67604957	0.9574579	0.01430009
CEBPG	0.7544265	0.58147642	0.9788177	0.03391186
TBCEL	1.2535945	1.00921078	1.5571565	0.04106882
CLK1	1.3820203	1.16309101	1.6421587	0.00023608
SEPT2	1.5560508	1.14059935	2.1228261	0.00526902
NBPF20	1.5181753	1.11725706	2.0629598	0.00761509
QRSL1	0.7505032	0.58596648	0.961241	0.02302448
ECI1	0.7970144	0.64508486	0.9847263	0.03550047
SOCS5	1.3292853	1.04779642	1.6863957	0.01905085
CETN2	1.2802153	1.03706702	1.5803716	0.02152538
KTN1	1.2015172	1.00242628	1.4401494	0.04701314
SLC5A3	1.2712192	1.05607282	1.5301957	0.01119165
TMEM181	1.2811881	1.02724136	1.5979137	0.02791911
CNTRL	1.250867	1.00310809	1.5598202	0.04686493
UQCR10	0.7673056	0.60476987	0.9735237	0.02919053
UHRF2	1.5333433	1.21719198	1.9316112	0.0002853
ZNF37BP	1.2880043	1.0454358	1.586855	0.01743824
EPS8	0.8036715	0.66512381	0.9710791	0.02357706
ACTR2	1.2574866	1.01109675	1.5639181	0.0394803
ZNF275	1.272054	1.0266798	1.5760722	0.0277521

PANK1	0.7908452	0.67787638	0.9226404	0.00284714
BNIP2	1.3106949	1.08824604	1.5786146	0.00435637
STOX1	0.8083736	0.68596977	0.9526191	0.01110452
ENDOG	0.8002918	0.67484683	0.9490553	0.01043504
GGH	0.8714894	0.7844881	0.9681393	0.01036595
EHBP1	1.3636643	1.10377956	1.6847388	0.00403685
SENP7	1.3644841	1.09603333	1.6986863	0.00543043
RBM17	1.4839001	1.03003757	2.1377468	0.03410319
ZNRF2	0.7618068	0.59652171	0.9728892	0.02924112
RPS25	1.5604205	1.19098953	2.0444446	0.00124683
TIGD1	1.2207112	1.01969632	1.4613525	0.02982321
B3GNT6	0.9054582	0.83115017	0.9864097	0.02301659
OCLN	0.7104663	0.58429364	0.8638846	0.00061095
OSBPL8	1.329755	1.09002967	1.622202	0.00495569
WNK1	1.3438401	1.04050103	1.7356121	0.02356594
CERS6	0.8280248	0.70145823	0.9774283	0.02576644
CCDC50	1.2988455	1.0501624	1.6064178	0.01589429
CYB561D2	0.7110371	0.52998893	0.9539327	0.02293515
ZNF354B	1.2848938	1.00501807	1.6427089	0.04551126
NDC1	0.8251448	0.68636535	0.9919847	0.04079399
LRCH3	1.7808247	1.24867289	2.5397657	0.00144206
TRIM2	0.8258762	0.70212168	0.9714435	0.02090174
GLCE	0.8086199	0.69991196	0.9342119	0.00392898
CTTNBP2	0.8883829	0.79702157	0.9902168	0.03255535
ZNF281	1.2816719	1.05267747	1.5604806	0.01346745
RAB3GAP1	1.4207095	1.02727078	1.9648331	0.03378763
SETX	1.4056562	1.07527242	1.8375523	0.01274351
SMS	1.2570341	1.00867906	1.5665386	0.04165888
ZNF333	1.8968475	1.24705363	2.8852252	0.00277411
STIL	0.8311488	0.71109116	0.9714766	0.02015125
NMD3	1.2693424	1.00593214	1.6017284	0.04445175
TSPAN15	0.8087397	0.66277721	0.9868472	0.03658717
TARS	1.3592344	1.03506959	1.7849217	0.0272496
NANS	0.7048251	0.5297356	0.9377855	0.01635831
SNRPA1	1.3657195	1.04591894	1.7833024	0.02203317
TMEM245	1.3027553	1.0262762	1.6537179	0.02977501
SPATA2	0.7600994	0.61796166	0.9349303	0.00940526
LINC00525	0.8320902	0.70030277	0.9886783	0.03667265
FFAR4	0.9056013	0.83026484	0.9877737	0.02524951
RNF20	1.350922	1.02149455	1.7865884	0.03493731
NUDT4	0.763835	0.61293706	0.9518823	0.01643458
ZNRF3	0.8319586	0.70029894	0.988371	0.03634544
MCM6	0.7837953	0.62573361	0.9817836	0.03401023
MIPEP	0.8106375	0.66346444	0.9904573	0.03999819
SON	1.3981969	1.06879977	1.829112	0.01446949
MECP2	1.6154216	1.20958189	2.1574288	0.00115831
DUSP16	0.7378359	0.59215343	0.9193594	0.00674536
SKA2	0.824309	0.69024174	0.9844165	0.03289279
AKAP1	0.7737001	0.60596406	0.9878668	0.03960206
MAP7	0.7961254	0.64356563	0.9848501	0.03567503
GNL3	0.7396503	0.59376625	0.921377	0.00713441
HNF4G	0.8272595	0.7142665	0.9581273	0.01137971
ARIH1	1.5372184	1.16209326	2.0334344	0.00259158
KDELC1	1.2270099	1.03187707	1.4590432	0.02060968

CENPH	0.8265059	0.69938552	0.9767318	0.02533434
PTRHD1	0.6780812	0.52875405	0.8695802	0.00220543
PDIK1L	0.7895048	0.66012872	0.9442367	0.00964422
FITM2	0.7584677	0.63844307	0.9010565	0.00165889
CHST5	0.8869793	0.79366957	0.9912591	0.03444976
DSTYK	1.4637954	1.10672386	1.9360718	0.0075688
TOPORS	1.2388593	1.00752521	1.5233091	0.04224949
GPR157	0.7987481	0.65157498	0.9791637	0.03057108
B9D2	0.7516838	0.57103369	0.9894838	0.0418159
MYB	0.7154501	0.61130583	0.8373368	3.02E-05
CELF1	1.700459	1.23448177	2.3423275	0.00115736
CABLES2	0.8317218	0.69867435	0.9901053	0.03828656
LIN54	0.7019409	0.53849786	0.9149917	0.00887396
BBX	1.2755478	1.04863737	1.5515585	0.01488554
INSIG2	1.2387439	1.0041637	1.5281238	0.04563494
GPR160	0.8961432	0.80859407	0.9931716	0.03656438
NOP14	0.7667067	0.60300195	0.9748546	0.03017537
TTBK2	1.4568989	1.05671813	2.0086286	0.02163817
CDK17	1.3304889	1.04840121	1.6884765	0.01883648
RCOR3	1.340892	1.01965484	1.7633334	0.035794
RBM25	1.2524807	1.01317307	1.5483119	0.03744004
RNF146	1.4423299	1.11489685	1.8659265	0.00530652
ABTB1	1.4137032	1.11411738	1.7938475	0.00438137
PIPOX	0.8883499	0.80676198	0.9781889	0.01601298
COPS2	1.2590162	1.0228933	1.5496454	0.02973776
TMEM54	0.8361522	0.72041368	0.9704847	0.01856777
STARD10	0.7359096	0.5999628	0.902661	0.00325349
ARMCX3	1.1800893	1.01746879	1.3687012	0.02860521
RDX	1.234443	1.08396201	1.4058146	0.00149571
FLT1	1.5623651	1.29304032	1.887787	3.80E-06
TEX2	0.7537825	0.56881123	0.9989045	0.04911475
COA7	0.6528561	0.49688887	0.8577794	0.00220322
FAM120C	1.3495511	1.07693325	1.6911803	0.00922169
AP3M2	1.3147534	1.04788929	1.6495794	0.01807474
ATOH1	0.8406004	0.77394866	0.912992	3.79E-05
THUMPD3	0.6819188	0.5124217	0.9074816	0.00864402
RNF149	1.564875	1.14928213	2.1307509	0.00446279
VTI1A	0.7071661	0.50854697	0.9833582	0.03942493
UXS1	1.4108666	1.02003739	1.9514427	0.03754001
MRS2	0.7394081	0.56308973	0.9709363	0.02984265
PLA2G12B	0.9040078	0.83724346	0.9760962	0.00993662
SERTAD1	1.2462311	1.01201104	1.5346592	0.03823105
CEP164	1.496165	1.12990993	1.9811401	0.00491462
TMEM97	0.7882799	0.67203945	0.924626	0.00346988
MYO9A	1.3831401	1.05564845	1.8122288	0.01863311
FKBP14	1.2482896	1.01319845	1.5379287	0.03723974
NOD1	1.3731062	1.04238424	1.8087578	0.02412017
RPL32P3	1.2860667	1.02735241	1.6099321	0.02813176
RASAL1	1.1639337	1.0254478	1.321122	0.01883541
SPAG1	0.8958893	0.80333106	0.9991118	0.04816226
PRKAR1A	1.3065495	1.0386555	1.6435397	0.02237625
PDPK1	0.6860731	0.52229504	0.9012076	0.00678047
ACSL5	0.8149172	0.6990657	0.949968	0.00889642
PRLR	0.7804219	0.65717183	0.9267871	0.00469994

YEATS4	0.8315913	0.70193921	0.9851909	0.03296645
NDUFB10	0.6042763	0.45852075	0.7963649	0.00034787
TCEAL4	1.3391427	1.12165434	1.598802	0.00123959
NAB1	1.3853381	1.14660193	1.673782	0.00073129
PANX1	1.3815542	1.08311167	1.7622302	0.00924303
PTPDC1	1.3259473	1.01617922	1.7301439	0.03769199
FAM214B	1.2573825	1.00024332	1.5806262	0.04975687
NPHP3	1.3614084	1.07496605	1.7241779	0.01047524
ALMS1-IT1	1.4241774	1.1440986	1.7728204	0.00155158
TXN2	0.6226836	0.46917003	0.8264272	0.00103823
DDX6	1.3135283	1.00812585	1.7114496	0.04339317
KDM2A	1.4210897	1.06989957	1.8875564	0.01524614
SNX7	0.8111511	0.66160894	0.994494	0.04410756
PLEKHM3	1.386108	1.05124023	1.8276465	0.02065992
ZFAND6	1.3346881	1.06488878	1.6728436	0.01222375
RBPJ	1.312966	1.01408746	1.6999321	0.03881782
MAML2	1.2994942	1.07378009	1.5726547	0.00711866
LCA5	1.5868562	1.19983073	2.0987232	0.00120732
NDUFB7	0.6985628	0.5444373	0.8963198	0.00479333
STAMBPL1	0.7948454	0.63455243	0.9956297	0.0457082
WSB1	1.3435057	1.08771819	1.6594441	0.00613914
KCNMB3	1.5002356	1.08060507	2.0828209	0.01539085
CREBRF	1.2558713	1.03604867	1.5223347	0.02030326
IGF1R	1.2402846	1.01111362	1.5213977	0.03882839
COPB1	1.4199021	1.02650108	1.9640719	0.03417692
ZBTB34	1.3532345	1.0599431	1.727681	0.01522236
VAV3	0.9158449	0.85141706	0.985148	0.01817586
HEPACAM2	0.9190999	0.86871182	0.9724106	0.00336258
SERINC1	1.3271867	1.08654923	1.621118	0.0055509
LMNA	1.4155688	1.09702709	1.8266048	0.00754159
TCF20	0.7542875	0.57139834	0.9957145	0.04656043
ITLN1	0.9191532	0.88091808	0.9590478	0.00010072
ACE2	0.9329933	0.87600119	0.9936933	0.03102945
COX17	1.3949034	1.0927842	1.7805487	0.00753089
ABAT	0.8954579	0.80198211	0.9998288	0.04964522
STX6	1.4144781	1.00512688	1.9905429	0.04666801
ZSCAN26	1.3071187	1.01739068	1.6793542	0.03618723
ARCN1	1.4330347	1.05567951	1.9452765	0.02102887
CACNA1D	0.8369259	0.70216865	0.9975451	0.04687768
SNRNP27	1.2882188	1.02851887	1.6134928	0.02747088
AP1S2	1.2759431	1.07137095	1.5195771	0.00627246
GIPC2	0.8209746	0.73681387	0.9147484	0.00035063
HIVEP2	1.5458819	1.19939366	1.9924658	0.00076779
CHPT1	0.7682127	0.63035121	0.9362254	0.00897566
ABL2	1.5185395	1.1955111	1.9288506	0.00061862
TOX3	0.8480865	0.73422029	0.9796117	0.02509114
MPP7	0.8064202	0.68712387	0.9464285	0.00843721
CCNA2	0.8320197	0.71978546	0.9617544	0.01286705
CDV3	1.413701	1.07209413	1.8641559	0.01415732
F2RL2	0.7912762	0.69156766	0.9053605	0.00065738
EVA1B	1.2878635	1.06987063	1.5502738	0.00750058
KLK11	1.0849001	1.00349313	1.172911	0.04060073
RAB11FIP4	0.6489992	0.51588562	0.8164599	0.00022305
SATB2	0.9055483	0.82809589	0.9902449	0.02964113

ASRGL1	0.7993785	0.71097069	0.8987796	0.0001807
NSMCE4A	0.6986522	0.53175207	0.9179369	0.01003089
ZXDC	1.507854	1.06576346	2.1333286	0.02035619
VAV2	1.2555532	1.05695048	1.4914738	0.00958573
NDUFA2	0.705523	0.50184847	0.9918584	0.04474963
NUP85	0.7325279	0.56016434	0.9579281	0.02296657
PLEKHA8P1	1.333589	1.05402162	1.6873084	0.01647209
PTP4A2	0.7121882	0.5331278	0.9513891	0.02160499
FBXO28	1.3458158	1.0290676	1.7600594	0.03006432
MTCH2	0.6247294	0.45904955	0.8502064	0.00277089
ST6GALNAC1	0.905401	0.84862358	0.9659771	0.00263357
FOXA3	0.8524651	0.74800509	0.9715131	0.01669857
VEGFA	1.2587721	1.04826407	1.5115534	0.01370932
LRRFIP1	1.3526895	1.05937354	1.727218	0.01541507
KBTBD8	0.7768153	0.63083527	0.9565762	0.01740801
UBR1	1.2643411	1.00370799	1.5926529	0.04643832
UACA	1.4859474	1.17152461	1.8847575	0.00109454
PAK1	0.6270412	0.46173227	0.851534	0.0027964
AURKAIP1	0.6891938	0.51061473	0.9302281	0.01498969
SEMA4B	1.2104554	1.0191797	1.437629	0.02952151
NIN	1.5705377	1.2619631	1.9545649	5.24E-05
PLA2G12A	0.76235	0.60496388	0.9606813	0.02145059
PSMG1	0.841735	0.72278949	0.9802547	0.02665521
ASNS	1.2553831	1.0412419	1.5135645	0.01714946
PIGR	0.944249	0.90328131	0.9870748	0.01125067
KLF2	1.2819938	1.1065989	1.4851886	0.00093516
MPND	0.767964	0.60824632	0.9696216	0.02646743
RCAN3	0.7642032	0.59950381	0.9741497	0.0298979
WWC3	1.3536235	1.12911052	1.6227789	0.00106676
ZNHIT3	0.7368926	0.55968973	0.9701994	0.02958973
HIP1	1.2219035	1.01188845	1.4755068	0.03727058
SDCCAG8	1.5839153	1.18147929	2.1234294	0.00210492
GLA	0.8136821	0.66475661	0.9959715	0.0455973
TEX264	0.7235737	0.5336583	0.9810752	0.03725439
TGFBR2	1.2479345	1.04501772	1.4902528	0.01443274
ZNF767P	1.2820004	1.05861401	1.5525253	0.01098858
AGR2	0.8981543	0.82192923	0.9814485	0.01760629
SLC25A48	0.5801563	0.42228784	0.7970425	0.0007799
PDE12	0.6533224	0.46533738	0.9172489	0.01393615
DUSP4	1.0890112	1.00515931	1.1798582	0.03699271
RETNLB	0.9277916	0.87214181	0.9869924	0.01755691
MIS18A	0.8227021	0.67969905	0.9957918	0.04515035
LGALS9	0.7570083	0.63954952	0.8960394	0.00121232
NDUFA6	0.7683398	0.5961868	0.9902031	0.04174734
LINC-PINT	1.2587963	1.03991181	1.5237525	0.01820026
KLK12	0.8596183	0.77619877	0.952003	0.00367992
HES4	1.1567776	1.0260755	1.3041287	0.01727699
METAP1	0.6839343	0.52000405	0.8995433	0.00658398
HNF4A	0.768558	0.63810746	0.9256771	0.00554181
XKRX	0.7990117	0.6939656	0.9199588	0.00180843
TCEA3	0.8630896	0.76105936	0.9787984	0.02180095
LGR5	0.915314	0.85334599	0.9817821	0.01336058
CEP19	1.4170615	1.07845868	1.8619751	0.01234461
MCM7	0.8249104	0.68510327	0.9932477	0.04220726

RFFL	0.5926973	0.42427188	0.8279835	0.00216491
CLDN7	0.7946869	0.65468668	0.9646252	0.02011382
ZNF852	0.6755245	0.47773407	0.9552037	0.02646958
CAMTA1	0.6500236	0.46419345	0.910247	0.01216354
AHCY	0.8385767	0.71508672	0.9833924	0.03031036
WDR47	1.3930039	1.1047703	1.7564375	0.00507321
WDPCP	1.5561651	1.04080508	2.3267083	0.0311738
AIDA	1.2619365	1.01875593	1.563165	0.03316326
EIF2AK4	1.3473662	1.03433448	1.755134	0.02709021
NUDT3	1.4963721	1.10478652	2.026753	0.00922141
CSGALNACT2	1.3509213	1.13220083	1.6118946	0.00084449
PHF13	1.3376141	1.02959116	1.7377884	0.02938033
TROVE2	1.330441	1.0423292	1.6981903	0.02185331
MARS2	0.7780466	0.63647894	0.9511021	0.01431643
MRPL27	0.6464866	0.48958238	0.8536764	0.00210268
KATNAL1	1.2760323	1.03844544	1.5679768	0.02040376
PDZK1IP1	0.8953042	0.81262162	0.9863996	0.02528979
SCRN1	1.2011636	1.0827778	1.3324932	0.00053574
LYL1	1.3164633	1.03439757	1.6754444	0.02542686
DUXAP10	1.2291609	1.01193344	1.4930197	0.03756924
FZD3	0.8584165	0.73908699	0.9970123	0.04559259
ABCC2	0.8719299	0.76983196	0.9875684	0.03101816
TSG101	1.4335608	1.02437929	2.0061872	0.03569043
EIF4G2	1.3939131	1.00315161	1.9368893	0.0478485
PEX11G	0.7708382	0.59617021	0.9966809	0.04710854
TP53BP1	1.3595307	1.05731002	1.7481379	0.01664712
PSMD6	0.643371	0.43459022	0.9524519	0.02757075
KLHL12	1.35445	1.01038185	1.8156847	0.04245448
INPP4B	1.2621034	1.02346195	1.556389	0.02949299
MUC5B	0.9144456	0.85328027	0.9799954	0.01133959
RRAGB	1.3614696	1.09387085	1.6945324	0.00571806
METRN	1.1858957	1.03224009	1.3624239	0.01603399
MAP4K4	1.5091903	1.22508433	1.8591825	0.00010985
MACF1	1.34743	1.10571825	1.6419802	0.00311419
IDS	1.4834645	1.18783947	1.8526636	0.00050512
ARFIP1	0.8276185	0.68877906	0.9944442	0.0434427
PLCB4	0.920523	0.86064453	0.9845674	0.0158142
RHOQ	1.2418129	1.01449532	1.5200654	0.0357751
RER1	0.7056016	0.51264844	0.9711793	0.03240459
HCFC1R1	1.5025396	1.17656436	1.9188285	0.00110209
LMNB1	0.8299078	0.69535045	0.9905033	0.03885806
SHC2	1.1983859	1.04353526	1.3762148	0.01035874
ROCK1	1.3119052	1.05447215	1.6321865	0.014856
ZNF57	0.762474	0.63432206	0.9165165	0.00387093
RNF207	1.2595767	1.01731549	1.5595294	0.03421893
OSBPL10	1.5454565	1.11811562	2.136126	0.00838866
RFC5	0.7551024	0.59860316	0.9525168	0.01776434
IL22RA1	0.8476827	0.74467751	0.9649358	0.01242096
PRR15	0.8631469	0.76581105	0.9728543	0.01591836
MEF2A	1.3854436	1.07981252	1.7775808	0.01035275
TST	0.7873982	0.66074201	0.9383329	0.00755515
COX4I1	0.6773791	0.4893902	0.93758	0.01884543
TMEM53	0.672711	0.52194489	0.8670266	0.00219827
CENPU	0.8287314	0.72043704	0.9533043	0.00855696

RNF157	0.7967829	0.694442	0.914206	0.00120029
ZWINT	0.7940212	0.6578304	0.9584077	0.01628478
LGALS4	0.7483076	0.66404655	0.8432605	1.97E-06
HACL1	0.6813483	0.53108559	0.8741255	0.00254225
ITGB1	1.4769579	1.1784467	1.8510847	0.00071103
CRYM	0.7768778	0.67562962	0.8932989	0.00039454
ISOC2	0.7663046	0.5955683	0.9859873	0.03848113
RRM2	0.8583529	0.74278881	0.9918965	0.03842926
TAF7	1.2693144	1.00773327	1.5987951	0.04282731
ECM1	1.1496501	1.00905231	1.3098383	0.03613866
AGMAT	0.6887707	0.57619441	0.8233421	4.23E-05
LRBA	0.7952177	0.64338634	0.9828794	0.03403017
NUDT9	0.7119454	0.53884118	0.9406598	0.01683214
NUDCD2	0.7148399	0.53243189	0.9597398	0.02552577
FAM168A	1.3129346	1.04693436	1.6465191	0.01842154
MTMR4	0.6898495	0.5315745	0.8952505	0.00523712
STX10	0.7118595	0.52601799	0.9633585	0.02767977
CLCA1	0.9619597	0.9269432	0.9982991	0.04036962
C2orf27A	1.4253907	1.120777	1.8127949	0.00385871
LINC00869	1.3636274	1.02015645	1.8227399	0.03619348
RORC	0.844745	0.73236345	0.9743717	0.02053589
GAR1	0.7329743	0.56132862	0.9571066	0.02248817
GATAD2B	1.4277821	1.0823298	1.8834941	0.01174381
VPS18	1.3263957	1.02037915	1.7241882	0.03479675
TSPYL4	1.2755169	1.05570389	1.5410982	0.01167957
IMPA2	0.7175016	0.60272861	0.8541298	0.00018931
RHPN2	0.6946266	0.57319778	0.8417794	0.0002017
FEM1B	1.2913536	1.01667851	1.6402374	0.03612425
UQCRQ	0.6274446	0.49409693	0.7967803	0.00013154
SF3B5	0.7379906	0.56547144	0.9631435	0.02532673
EXOC6B	1.3964985	1.00321594	1.9439565	0.04781713
YLPM1	1.3329369	1.0144487	1.7514151	0.0391184
GNG10	1.3852524	1.11959501	1.7139449	0.00270097
TSC1	1.3177289	1.00347207	1.7304015	0.04715573
COX6A1	0.6673259	0.48408914	0.9199213	0.01352706
DENND6B	1.3908305	1.03752075	1.8644538	0.02736294
POGZ	1.4267409	1.1119789	1.830601	0.00519648
ANGPT2	1.3492235	1.14041377	1.5962664	0.00048018
NXF1	1.4096425	1.00764533	1.9720151	0.04502419
GYG2	0.8713276	0.7711103	0.9845697	0.02714638
ACVR1	1.3547259	1.05093842	1.7463271	0.01910536
ADAMTS6	1.52658	1.18590195	1.9651258	0.00102594
SPACA3	0.8474035	0.73352438	0.9789622	0.02452982
GTF2H5	1.4260907	1.02097048	1.9919622	0.03737146
RAB7A	1.6410301	1.07109936	2.5142202	0.02287576
MSRB2	1.2623824	1.002737	1.5892594	0.04734189
FOLR2	1.1543359	1.01742858	1.3096659	0.02586655
GCAT	0.7933525	0.65963741	0.954173	0.01396787
DBI	0.6636641	0.52210775	0.8435999	0.00080967
SYNGR1	1.3299506	1.07423976	1.6465306	0.00886293
SHQ1	0.6200932	0.45001708	0.8544466	0.0034817
EDA	0.8085872	0.67803706	0.9642737	0.01803497
TRPM4	0.8472732	0.73817177	0.9724997	0.01845043
GYPC	1.2083015	1.03923542	1.4048718	0.01387852

SLCO3A1	1.2134295	1.02400394	1.4378959	0.02549126
ACY3	0.8154976	0.68271959	0.9740989	0.02448803
AARS	1.419424	1.09630512	1.8377772	0.00786932
TMEM154	0.8372874	0.70453304	0.9950565	0.04377609
TBXA2R	1.444413	1.09341235	1.9080898	0.00963474
RAB18	1.3747416	1.04017861	1.8169135	0.02529811
DYNC2H1	1.436648	1.08641364	1.8997897	0.0110438
DDC	0.8613381	0.78978993	0.939368	0.00074189
SUPT3H	1.4398936	1.07992626	1.9198474	0.01299739
CCL28	0.9031798	0.82094861	0.9936477	0.03654587
CLPP	0.7654475	0.59037492	0.9924369	0.0436685
MYRIP	0.8744101	0.76476581	0.999774	0.0496148
SNAPC1	1.3355686	1.08794065	1.6395596	0.00568297
GSS	0.6847715	0.53880518	0.8702812	0.00196227
IGIP	1.2812351	1.02773149	1.5972687	0.02758502
TJP1	1.323043	1.07200171	1.6328731	0.00911735
INTS12	0.6918964	0.52676879	0.908787	0.00811015
TJAP1	1.6646663	1.23449497	2.244735	0.00083466
FAR2	0.7695468	0.64403705	0.9195159	0.00393108
VIL1	0.8008223	0.70432798	0.9105365	0.00069732
RANBP1	0.7342145	0.55202411	0.9765353	0.03374246
SMIM22	0.8055136	0.66277804	0.9789887	0.02975343
ZBED3	0.7495972	0.62742869	0.8955534	0.00149696
AMACR	0.7281198	0.60047081	0.8829045	0.00125397
SLC27A1	1.4309948	1.17209974	1.7470749	0.00043241
KANSL1L	1.3728437	1.0801387	1.7448684	0.00959606
MLH3	1.448632	1.12851805	1.8595491	0.0036267
TSPAN4	1.1650837	1.00032843	1.3569744	0.0495086
RIMS3	0.8129038	0.68224569	0.9685844	0.0205036
SLC20A1	1.2698803	1.07079634	1.5059783	0.00602925
PPIC	1.2692903	1.05981972	1.5201623	0.00956074
OAT	1.1905254	1.0199295	1.3896556	0.02710243
ETS2	0.7759045	0.65393998	0.9206164	0.00363851
SLC18A1	0.787729	0.67740061	0.9160265	0.00194011
PLA2G2A	0.9485734	0.9068161	0.9922536	0.02153214
TM2D3	1.6664534	1.22480262	2.2673588	0.00115121
ARL15	0.8112932	0.6675188	0.9860347	0.03561683
EMP3	1.1449388	1.00983501	1.2981179	0.03462379
RRN3P2	1.3416262	1.08518796	1.6586627	0.00662115
CDC37L1	1.2946399	1.03501117	1.6193956	0.02374025
GMDS	0.8193458	0.68880288	0.9746293	0.02443753
PHF21A	1.5989013	1.16076621	2.202412	0.0040738
ITPKA	0.8589769	0.75401258	0.978553	0.02225456
RALY	0.7853193	0.63054089	0.978091	0.03094669
RNH1	1.6684751	1.19043704	2.3384766	0.00295841
SELPLG	1.1808424	1.01385115	1.3753388	0.03261484
STC2	1.1294546	1.01853498	1.2524535	0.02098892
HMGCL	0.7444794	0.57288094	0.9674777	0.02729357
CAPRIN2	1.3502231	1.11472723	1.6354696	0.00213605
UBE2H	1.5493277	1.15293715	2.0820011	0.00368598
FAM129B	1.3632848	1.05400317	1.7633206	0.01824523
PKD2	1.1780015	1.02875135	1.3489048	0.01778536
GINS3	0.7999148	0.65781177	0.9727154	0.02527449
ARPP19	1.3127068	1.05318	1.6361868	0.01547823

CDC25C	0.7083487	0.56143031	0.8937134	0.00364392
PLA2G3	0.8700324	0.76157347	0.9939375	0.04041522
FARP2	0.6517808	0.47230799	0.8994516	0.0091919
LINC00261	0.887293	0.8220645	0.9576971	0.00214447
SGIP1	1.3212538	1.06489646	1.6393252	0.01136428
BICD2	1.5097277	1.16547131	1.9556704	0.00181091
TFB1M	0.6620011	0.47665542	0.9194178	0.01384463
C9orf78	1.463932	1.06338433	2.0153551	0.01945125
DFFB	0.7484478	0.60084693	0.9323076	0.00972729
RAMP1	1.0777824	1.00251855	1.1586968	0.04255331
REEP4	0.7353271	0.58751624	0.9203251	0.00725047
DCAF8	1.4372492	1.02288951	2.0194608	0.03658303
ZBTB43	1.3947085	1.03735439	1.8751661	0.02760965
KIAA1324	0.9128954	0.83919927	0.9930634	0.03383398
ELP6	0.6381232	0.44095794	0.9234468	0.01720388
MAGI3	0.7627726	0.59659119	0.975244	0.03077989
LINC01138	1.4461088	1.05952968	1.973735	0.02010805
STARD5	0.7882557	0.64363075	0.9653781	0.02141028
CHSY1	1.264607	1.06174623	1.5062271	0.00849868
HSF2BP	1.3757168	1.02352373	1.8490991	0.03450959
LINC01278	1.3062106	1.05436761	1.618208	0.01450898
ESM1	1.2701711	1.09499224	1.4733754	0.00158607
COX11	0.6746739	0.52386113	0.8689038	0.00229935
SLC35G1	0.7038005	0.55803972	0.8876341	0.00301051
PAXBP1-AS1	1.6711965	1.11531795	2.504127	0.01281322
ACOT9	1.3871575	1.0786538	1.7838958	0.01077513
HAS2	1.1502013	1.020482	1.29641	0.02190259
CKMT2	0.8995416	0.83916352	0.9642639	0.00282189
MFSD2A	0.8188932	0.67152939	0.9985952	0.04839862
PJA1	1.2524727	1.0508058	1.4928427	0.01196356
GJB5	1.0940026	1.00772542	1.1876665	0.03206677
ARMCX4	1.4063968	1.08645181	1.8205612	0.00960909
ZNF83	1.2156518	1.04931553	1.4083556	0.00929058
MOGAT2	0.898853	0.81481335	0.9915604	0.0332383
RPS6KA5	0.7375042	0.56740735	0.9585925	0.02284087
ZNF516	1.3026939	1.01390907	1.6737313	0.03864103
ZSCAN9	1.4255555	1.07595073	1.8887561	0.01351448
COL4A1	1.2162035	1.0745381	1.3765458	0.00195014
CTXN1	1.1407669	1.00251054	1.2980903	0.04571633
RABGAP1	1.5988023	1.24877491	2.0469413	0.0001975
EFCAB11	0.7306269	0.53431989	0.9990563	0.04931272
GZMB	0.8987271	0.83204243	0.9707562	0.00663763
TRPC1	1.4006432	1.15598648	1.6970798	0.00058204
SMDT1	0.7282571	0.53766867	0.9864038	0.04052133
KLHL20	1.3751915	1.0287541	1.8382931	0.03144499
FHDC1	0.7303527	0.62149322	0.8582798	0.00013576
APEH	0.731707	0.54207947	0.987669	0.04124741
KIAA1024	1.5941842	1.14762149	2.2145134	0.00541815
CHD3	1.2890729	1.05562663	1.5741446	0.0127372
PLD3	1.234837	1.00305038	1.5201852	0.04673671
PRRX2	1.3845795	1.20645877	1.5889978	3.63E-06
KIF16B	0.8375807	0.70612911	0.993503	0.04187128
COX7A1	1.194551	1.04431314	1.3664025	0.00953574
GPRASP1	1.2867326	1.11670262	1.4826516	0.00048949

PMCH	0.7076489	0.50310609	0.9953507	0.04695146
FGD1	1.1932216	1.03076071	1.3812883	0.01799676
LARS2	0.6830145	0.54538671	0.8553726	0.0008981
CCNB1	0.8029003	0.69985517	0.9211176	0.0017337
KCTD1	1.3323992	1.08541386	1.635586	0.00607873
POC1B	0.8014786	0.68338375	0.9399813	0.00650821
COX5A	0.6226883	0.48399037	0.801133	0.00022905
PSMB10	0.7667969	0.6424378	0.9152286	0.00327035
AGAP6	1.4045117	1.10820885	1.7800374	0.00495642
CARS	1.5945931	1.14254013	2.2255035	0.00608048
PTGER4	0.858671	0.74189161	0.9938323	0.04106097
DHFR	0.8214972	0.68708807	0.9821995	0.03100613
MRPS34	0.6612147	0.5192198	0.842042	0.00079703
PRNP	1.2649809	1.09861674	1.4565378	0.00108586
FXYD3	0.8224951	0.73608131	0.9190537	0.00055974
ELK3	1.3517707	1.12225503	1.6282252	0.00149908
PPM1M	1.371008	1.11648475	1.6835544	0.00259901
SEMA5A	0.8854884	0.78634941	0.9971264	0.0447001
RPP14	0.6009644	0.44180731	0.8174564	0.00117868
TMEM39A	1.8024609	1.26496068	2.5683527	0.00111065
NFATC1	1.3019213	1.08171654	1.5669531	0.00525724
TJP3	0.8159957	0.68334444	0.9743974	0.02467224
FUT4	0.8255557	0.7046855	0.967158	0.0176249
CYCS	0.8285965	0.69025252	0.9946681	0.04366166
PSMA5	0.6732349	0.51837629	0.8743556	0.00300988
VPS9D1	1.5190161	1.15605297	1.9959378	0.0026922
BMP2K	0.7361587	0.5486259	0.9877944	0.04116884
LINC00899	1.39828	1.04859398	1.8645797	0.02242356
SACS	1.1629442	1.02249661	1.3226832	0.02151932
IDUA	1.3386447	1.07980872	1.6595252	0.00780604
TPD52	0.8278724	0.70920999	0.9663889	0.01670675
SLC16A6	1.3061701	1.04119785	1.6385745	0.02094571
WDFY2	1.4594185	1.0888031	1.9561868	0.01143351
GTSE1	0.7690728	0.61356199	0.9639987	0.02272206
FAM104A	0.6503262	0.4767878	0.8870282	0.00658948
TTPA	0.8305574	0.72563885	0.9506459	0.00704853
BTBD10	1.4390391	1.04655868	1.9787074	0.02508885
MCCC2	0.6467365	0.50042739	0.8358218	0.00086703
HMCN1	1.1695404	1.01854007	1.3429268	0.0263905
ADCY4	1.2737906	1.01776074	1.5942278	0.03453796
IFITM10	1.2926981	1.03442805	1.6154515	0.02396944
TRAPPC6B	1.2445973	1.01362944	1.528194	0.036688
CLCN3	0.7723168	0.63908126	0.9333293	0.00749292
ABCD3	0.7947813	0.66345082	0.9521088	0.01268381
SPG11	1.3390983	1.0000054	1.7931746	0.04999576
DDAH1	0.7548291	0.58531593	0.9734349	0.0302009
KIAA1671	0.7288283	0.56762647	0.9358102	0.01313334
COX14	0.7295144	0.54726132	0.9724626	0.0315269
PPP1R8	0.7013396	0.51151774	0.9616034	0.0275871
CYP24A1	1.2267579	1.05225298	1.4302026	0.00903947
PHLDA3	1.2037636	1.05707486	1.3708081	0.00515572
ZG16	0.9496114	0.90732148	0.9938724	0.02612143
RAPGEF3	1.3771353	1.03589522	1.8307852	0.02761502
NDUFAF2	0.8142956	0.66643211	0.994966	0.04450262

CDON	1.3149514	1.01137057	1.7096575	0.04091545
RPS6KA1	0.7001564	0.57223797	0.8566698	0.00053442
CALU	1.3117226	1.07825976	1.5957344	0.00665842
LRRC34	1.2536071	1.06764435	1.471961	0.00579926
FSCN1	1.1713128	1.0674026	1.2853385	0.00084942
RAB11FIP1	0.7986519	0.6812051	0.9363476	0.00559934
TAF6	1.3659587	1.02719603	1.8164431	0.03199449
PLS1	0.8602659	0.74211505	0.9972273	0.04584706
ACVR1B	0.7501782	0.57980811	0.9706097	0.02874822
CASP6	0.7148256	0.59051507	0.8653049	0.00057274
WAS	1.2229293	1.03166231	1.4496567	0.02038522
LRRFIP2	0.731403	0.53748507	0.995284	0.04658664
DTX1	1.2836631	1.02728227	1.6040295	0.02803842
TNFRSF11A	0.803429	0.72138047	0.8948095	6.83E-05
VSIG10	0.7949273	0.64382858	0.9814871	0.03286813
WDR45B	0.7260577	0.54202642	0.9725721	0.03183817
ZNF25	1.3267982	1.07249871	1.6413944	0.00919612
SMPDL3B	0.7692386	0.6459096	0.9161158	0.00325409
STAB1	1.1949244	1.01205099	1.4108424	0.03561367
STAP2	0.7908571	0.66424842	0.941598	0.00838864
KHK	0.7270739	0.60158467	0.87874	0.00097648
GRSF1	0.7014958	0.52540837	0.9365979	0.01621122
TMEM252	0.8749826	0.78066873	0.9806907	0.02173119
SGSM2	1.2689874	1.00275984	1.605897	0.04737806
FAM167B	1.329246	1.09219843	1.6177417	0.00451148
SPG21	1.9116641	1.32475618	2.7585905	0.00053437
CD3D	0.8645437	0.76260577	0.9801076	0.02297451
ETV2	0.7121058	0.52330751	0.9690185	0.03075747
WNT4	0.8090924	0.6765967	0.9675342	0.02025043
CYTH3	1.3891304	1.07224168	1.7996719	0.01284806
NES	1.1761098	1.02101122	1.354769	0.02456677
ZNF442	1.6824844	1.0498129	2.6964365	0.03062055
FUT6	0.8810418	0.77707711	0.9989159	0.04805351
SCML4	0.7759246	0.63480305	0.9484185	0.01324763
SLC44A4	0.9017449	0.81854844	0.9933974	0.0362516
KDR	1.283847	1.09512268	1.5050945	0.00206935
CRIP2	1.2374069	1.08248787	1.414497	0.00179986
ATP10B	0.8736041	0.784779	0.9724828	0.0135113
SLC9A3R2	1.2284751	1.00035658	1.5086132	0.04960371
EDEM2	0.7303839	0.56240492	0.9485348	0.01846248
MRPS35	0.7082875	0.56194237	0.8927449	0.00349229
TERF2IP	1.784683	1.33828791	2.3799762	8.01E-05
IRF8	0.8095383	0.70597429	0.9282948	0.00248362
ANGPTL4	1.2597462	1.1118177	1.4273566	0.00029109
HDHD3	0.7136511	0.5764972	0.8834353	0.00194743
CABYR	1.3863306	1.00886645	1.9050217	0.04396763
LINC00852	0.5971887	0.44578977	0.8000057	0.00054881
SLC9A2	0.858244	0.75531308	0.9752018	0.01901684
SERTAD2	1.392495	1.09768617	1.7664816	0.00637462
FA2H	0.8170032	0.69142751	0.9653858	0.01761132
PHGR1	0.8735279	0.79111916	0.9645209	0.00748476
PTPRD	0.880547	0.79939673	0.9699353	0.00991532
RBP2	0.9201098	0.84949376	0.996596	0.0409875
CDH6	1.3942053	1.01957641	1.9064862	0.03739834

ACADSB	0.7597153	0.63603494	0.9074459	0.00243556
ABHD4	1.4591308	1.15465929	1.8438882	0.00155466
WDR26	1.3912014	1.03396952	1.8718553	0.02921384
SAV1	1.4068465	1.15455246	1.7142722	0.00071138
HSD17B7	0.761089	0.59637917	0.9712888	0.02822912
LAMC1	1.4776636	1.22457954	1.7830527	4.63E-05
TMEM238	0.8110709	0.6978198	0.9427019	0.00635405
C5orf30	0.7764612	0.65944196	0.9142457	0.00239987
ANKRD9	0.8064901	0.65357342	0.9951848	0.04496697
TBC1D14	0.7777501	0.61175254	0.9887906	0.04017038
SLC24A1	1.4921208	1.10866846	2.0081969	0.00827478
RTF1	1.3740692	1.02040442	1.8503115	0.03634889
EGFL7	1.2562916	1.06119495	1.4872561	0.00805528
PSCA	1.1049529	1.01128004	1.2073025	0.02723465
GRB10	1.2391642	1.03103929	1.489301	0.02226614
PRPF40B	1.431418	1.07676659	1.9028798	0.01354379
PELI1	1.2949053	1.05964079	1.5824039	0.01152939
NUDT6	0.8000407	0.6511357	0.9829982	0.03374037
SYCP3	0.6043227	0.36597986	0.9978852	0.04904075
L1CAM	1.145934	1.03573434	1.2678585	0.00827665
ABCC10	1.4361913	1.12085196	1.8402479	0.00421027
APLP2	1.4266876	1.084902	1.8761488	0.01098556
COX5B	0.7021588	0.54334063	0.9073995	0.0068779
EFNA5	1.1987767	1.02924405	1.3962339	0.01978079
CDH17	0.8981737	0.81310693	0.99214	0.03439579
CEBPA	0.7712438	0.67794007	0.8773888	7.87E-05
ABLIM3	1.2426853	1.07821227	1.4322474	0.00270356
PLXNA3	1.2505866	1.01185391	1.5456449	0.03854623
COX4I2	1.3482512	1.07923335	1.6843264	0.00850159
PDLIM7	1.3992399	1.17452049	1.6669547	0.00016934
HNRNPA3P1	0.4894703	0.26498309	0.9041376	0.02249964
KIF3C	1.4238357	1.14947134	1.7636874	0.00121438
STARD4	0.87349	0.76309851	0.9998509	0.04974762
RNF115	1.4362053	1.03290468	1.9969757	0.03136083
RIPK3	0.7726163	0.60264186	0.9905318	0.04184992
RAVER2	0.7304804	0.58754165	0.9081937	0.00470279
AHR	1.2726013	1.07116561	1.5119176	0.00610821
LINC00324	0.7813205	0.62780467	0.9723753	0.02703914
CRLS1	0.8087206	0.6601384	0.9907453	0.04039058
PMM2	0.6311767	0.49130481	0.8108694	0.00031803
C11orf68	1.5864122	1.18663924	2.1208666	0.00183871
BOK	1.2096567	1.04492616	1.4003567	0.0108236
GTF3C1	1.3635052	1.01883323	1.8247799	0.03702771
NAGK	1.3375992	1.0283519	1.7398438	0.03013007
SOWAHA	0.8599082	0.77674018	0.9519812	0.00363568
IER5	1.2525484	1.0376435	1.511962	0.01904048
LINC00622	1.3745995	1.09925006	1.7189208	0.00527617
ALDH1L2	1.3732705	1.1598308	1.6259889	0.0002329
PBX3	1.195045	1.02077556	1.3990661	0.02671521
INHBA	1.1454515	1.03325587	1.2698298	0.00982352
MAP3K8	1.2814789	1.07892727	1.5220564	0.00472242
NDUFA9	0.5378831	0.41494715	0.6972412	2.82E-06
PHF2	1.4438012	1.06579349	1.9558778	0.017722
FAM102B	0.8402854	0.71443634	0.9883029	0.03554464

TSPAN12	0.8342118	0.72470549	0.9602649	0.01158017
F8	1.2097488	1.01536293	1.4413487	0.03312583
CHPF	1.4352319	1.19650723	1.7215864	9.91E-05
NUDT16L1	0.737373	0.58022044	0.9370902	0.01272819
SECISBP2L	1.2499214	1.03428858	1.5105103	0.02094884
LRRC37A4P	0.7656183	0.60437234	0.9698846	0.02687119
CEACAM6	0.9189843	0.84872164	0.9950639	0.03735148
ZEB1-AS1	1.7822933	1.49114342	2.1302911	2.15E-10
ALDH3B1	1.4681695	1.18949211	1.8121361	0.00034923
TUSC2	0.6913768	0.48746985	0.9805772	0.0384551
HSPB2	1.3464571	1.0714041	1.6921223	0.01072485
PPP3CB	1.351451	1.02464358	1.7824929	0.03298026
SLC12A2	0.8163037	0.72404894	0.920313	0.00090956
ZNF532	1.3462574	1.17004365	1.5490097	3.27E-05
TNFSF11	0.8574756	0.7450704	0.9868388	0.03197191
NDEL1	1.5112008	1.06447358	2.1454059	0.02092005
ZMAT1	1.3149907	1.09163201	1.5840508	0.00393846
GNPTG	1.6486107	1.20660938	2.2525245	0.00169322
TMEM19	0.7694285	0.61685626	0.9597377	0.02010393
LIG1	0.7885869	0.63222328	0.983623	0.03516894
GGT6	0.8834356	0.81408815	0.9586903	0.00296435
ARL10	1.455969	1.0852188	1.9533809	0.01223238
EIF3K	0.6673217	0.47208554	0.9433001	0.02199198
ENPEP	1.198977	1.03463606	1.3894217	0.0158365
SDHAF1	0.7264732	0.53137344	0.9932061	0.04521082
SPHK1	1.1748724	1.05034002	1.3141699	0.00481597
FBL	0.7709853	0.623668	0.9531007	0.01621929
EPB41L4B	0.7706522	0.63689056	0.9325069	0.00739775
PDSS1	0.6951172	0.525429	0.9196064	0.01086839
UBE2E3	1.3391655	1.00653035	1.7817289	0.04500118
CALHM2	1.2181363	1.03609051	1.4321685	0.01688223
BCKDHB	0.7938251	0.6411306	0.9828861	0.03414662
SIDT1	0.8513801	0.75338515	0.9621215	0.00991196
SLC22A15	1.1954755	1.02661321	1.392113	0.02156059
AKAP8L	1.4770737	1.04945297	2.0789372	0.02530233
LIN52	1.3949248	1.0613131	1.8334035	0.01700203
NDUFS3	0.6889856	0.50353182	0.9427431	0.01988572
LOXL1	1.1578608	1.03547083	1.294717	0.01012658
MCU	0.8027487	0.65134691	0.989343	0.03935646
TRAIP	0.7641454	0.59451565	0.9821746	0.0356932
PWAR6	1.1723067	1.01924996	1.3483473	0.02594218
NLE1	0.8048176	0.65055118	0.9956655	0.04550314
RECK	1.2968518	1.02587579	1.6394037	0.02973654
SWAP70	1.3487208	1.0778921	1.6875974	0.0089011
NAT1	0.7998143	0.69420275	0.921493	0.00199131
FAM83A	1.2550269	1.04616377	1.5055888	0.01444921
TM4SF18	1.1972788	1.00501302	1.4263262	0.04380497
SYBU	0.7928013	0.69313796	0.9067949	0.0007057
ANKDD1A	1.5141204	1.1149683	2.0561665	0.00788426
GSTM4	0.8223222	0.69417569	0.9741249	0.02361915
PYY	0.8888805	0.79701298	0.9913371	0.03432088
IGFBP6	1.1945337	1.06628127	1.3382124	0.00215906
IL7	0.8144504	0.71665581	0.9255901	0.00166244
GMNN	0.7235634	0.60403286	0.8667475	0.00044424

PGP	0.7546776	0.5904085	0.9646512	0.0246202
TGFB2	1.4368478	1.21182647	1.7036529	3.03E-05
SLC4A4	0.8887609	0.80539604	0.9807547	0.01894222
SIPA1	1.4315569	1.13312917	1.8085802	0.00263148
PLEKHF1	1.2181611	1.01317126	1.4646255	0.03580337
SMARCA2	1.2679317	1.00507395	1.599535	0.04521369
RGS17	1.2960074	1.02118301	1.6447934	0.03297803
SLC26A2	0.9272445	0.86493703	0.9940404	0.03330517
SPRY4	1.4820441	1.19609255	1.8363585	0.00032172
CD177	0.9156398	0.84999108	0.9863589	0.02024332
DAPK1	1.2726983	1.12285399	1.4425393	0.00016131
BDH1	0.7565941	0.58811547	0.9733371	0.02998915
ERAP1	0.7567937	0.62222651	0.9204634	0.00527679
PPFIBP1	1.2589532	1.02131252	1.5518884	0.03096304
MARVELD2	0.661143	0.52609809	0.8308529	0.00038593
TGFBR1	1.2517661	1.03454781	1.5145926	0.02093021
TRIM8	1.660361	1.24527903	2.2138	0.00055135
KLHL21	1.2511865	1.01791108	1.5379217	0.03328814
ZNF641	1.9947767	1.4403368	2.7626415	3.24E-05
PHF1	1.6003504	1.24902341	2.0504991	0.00020057
BRD2	1.4009385	1.09509407	1.7922011	0.00730019
GUCY2C	0.8852201	0.80815488	0.9696343	0.00870277
ALG12	0.7083238	0.54329159	0.9234869	0.01083063
DMXL2	1.2410232	1.03196539	1.4924324	0.02177476
TBC1D19	1.2819144	1.00404632	1.636682	0.04633168
TGFB1	1.1677477	1.01144107	1.3482098	0.03441928
RNF34	0.5743649	0.39501265	0.8351507	0.00369445
ARHGAP29	1.3500379	1.09670146	1.6618948	0.00464763
PARVG	1.262828	1.02259169	1.5595026	0.03019918
OTOGL	1.9633537	1.15495681	3.3375772	0.01269825
SLCO5A1	0.7814155	0.64395445	0.9482196	0.01246943
CD82	1.265691	1.03421102	1.5489814	0.02223157
FAM162A	0.745809	0.57723084	0.9636199	0.02486877
HMGCR	0.7610803	0.63474339	0.9125628	0.0031991
GSR	0.711574	0.60107741	0.8423832	7.75E-05
USP30-AS1	0.8027143	0.68611106	0.9391341	0.00606742
TMEM144	0.7234462	0.57303598	0.9133359	0.00648375
NEK9	1.8436528	1.37065255	2.4798814	5.25E-05
GINS2	0.7514871	0.60080486	0.9399606	0.01234056
PPP4R1L	1.3203104	1.02509625	1.7005422	0.03140354
CALD1	1.1714869	1.04202985	1.3170271	0.0080721
COPZ2	1.3117421	1.12098627	1.5349585	0.00071335
GALK1	0.7084535	0.57026957	0.8801211	0.0018491
ULBP2	1.1495352	1.03172701	1.2807954	0.01153213
NOX1	0.889408	0.82854818	0.9547381	0.00119224
ORC1	0.7375081	0.62295653	0.8731238	0.00040725
DENND5A	1.3121114	1.11496176	1.5441214	0.00107579
SIMC1	0.7029227	0.53778614	0.9187673	0.00987832
TMEM88	1.4206354	1.14966224	1.7554765	0.00114765
IQGAP1	1.3191582	1.05143988	1.6550432	0.01669401
SEMA6B	1.2215134	1.00307228	1.4875249	0.0465381
PTTG1	0.8068078	0.68573259	0.9492605	0.0096635
TNFRSF19	1.1652048	1.0419167	1.3030814	0.00737109
VCAN	1.138257	1.03588374	1.2507474	0.00707791

SNX3	1.3844082	1.0290875	1.8624131	0.03159997
LGALS2	0.8864074	0.81485802	0.9642393	0.00498491
ACOX1	0.6890481	0.52826105	0.898774	0.00601135
GP2	0.8426797	0.73060432	0.9719475	0.01873672
COL8A1	1.1602076	1.04343684	1.2900462	0.00604039
COL5A2	1.14174	1.03910619	1.2545112	0.00581242
TSPAN14	1.3955688	1.02338938	1.9030999	0.03519967
TRAPPC3L	0.2916285	0.12101127	0.7028037	0.00603601
CACNA2D1	1.2836803	1.08391503	1.5202621	0.00380804
HSD17B4	0.7237087	0.55323183	0.9467175	0.01830016
SLC2A8	0.82909	0.68948635	0.9969598	0.04633482
NAGPA	0.6962691	0.53456256	0.9068922	0.00725851
BACE1	1.5864075	1.29315457	1.9461623	9.63E-06
HECTD2	1.4113872	1.03706226	1.9208239	0.02842222
GULP1	1.2550544	1.07761429	1.4617117	0.00348788
MSLN	1.1192701	1.05330539	1.189366	0.00027728
SLC11A1	1.3917166	1.18384474	1.6360888	6.21E-05
POU4F1	1.6629301	1.26340225	2.1888014	0.00028593
RPS27	1.4578406	1.13406822	1.8740489	0.003263
COL12A1	1.113248	1.00164105	1.2372906	0.04654852
OSR1	1.2435582	1.03913042	1.4882029	0.01736598
TMEM17	1.3270813	1.07160066	1.6434712	0.00949138
ADAMTS5	1.2700448	1.06831078	1.5098732	0.0067547
BRI3BP	0.7363499	0.61360897	0.8836429	0.00100338
PTGIR	1.4540762	1.13304737	1.8660628	0.00326768
VWA1	1.3030261	1.04897499	1.6186058	0.01675378
GRIPAP1	1.3388943	1.01688832	1.762866	0.0375914
PRDM11	0.6942299	0.496551	0.9706057	0.0328054
FJX1	1.2972005	1.12393073	1.4971822	0.00037502
SERPINI1	1.1999094	1.06368739	1.3535769	0.00303502
MFSD9	0.7330467	0.56430244	0.9522509	0.01999152
CCDC88A	1.200074	1.00182616	1.4375525	0.04772951
CEBPE	0.7256269	0.54659957	0.9632908	0.02650774
ILVBL	0.5946575	0.45549317	0.7763398	0.00013284
BIRC5	0.7855081	0.66689887	0.9252123	0.00384402
CKB	0.9201097	0.85960021	0.9848786	0.01644116
CCNF	0.73464	0.59194216	0.9117375	0.00513355
SEZ6L2	1.1979393	1.02870227	1.3950184	0.02011888
TRNP1	1.1055577	1.00068156	1.2214255	0.04845432
UGCG	1.2755832	1.04140073	1.5624268	0.01867528
C6orf89	1.4596674	1.02864511	2.0712963	0.03416368
SFXN3	1.3028217	1.04504329	1.6241856	0.01869125
EIF4G1	1.3709802	1.04161176	1.8044983	0.02439886
SLCO1C1	2.5019181	1.20980028	5.1740723	0.013372
TNNT1	1.1168378	1.02795059	1.2134112	0.00901591
SRPX	1.1135647	1.01289267	1.2242425	0.02608555
USP11	1.3159202	1.07921815	1.6045374	0.00665808
ADAM8	1.327503	1.14674529	1.536753	0.00014856
CLDN12	0.8069861	0.67585793	0.9635554	0.01777313
TRAP1	0.6683097	0.50806946	0.8790881	0.00395985
LEF1	1.2499405	1.07611837	1.4518397	0.00349789
MEIS3	1.328685	1.08624653	1.625233	0.0056957
LYPD5	0.7761746	0.63582495	0.9475046	0.01277967
SLC37A4	0.7554093	0.5934232	0.9616124	0.02273601

DNASE1L3	0.7979993	0.69803113	0.9122844	0.00095213
CERS5	1.6453375	1.07362135	2.5214995	0.02224833
HLA-F-AS1	1.437275	1.06665233	1.9366754	0.01712417
CYTH4	1.2729523	1.05703448	1.532975	0.01093241
AMIGO2	1.1193365	1.01565206	1.2336056	0.02301928
FEZ2	1.3515986	1.018397	1.793818	0.03696126
HOXB4	1.3210405	1.12423176	1.5523027	0.00071783
CCDC102B	1.2799615	1.08486788	1.5101391	0.00344018
ABCA3	1.1317551	1.00105411	1.2795209	0.04806528
WHAMM	1.4145238	1.069504	1.8708462	0.01505735
EP300-AS1	0.8633849	0.74681751	0.9981467	0.04714097
CRELD1	1.4196283	1.06771193	1.8875359	0.01592064
DBN1	1.2835994	1.12231244	1.4680648	0.00026816
ATF6B	1.4096312	1.03218239	1.9251056	0.0308371
PKHD1	2.0437568	1.19980006	3.4813648	0.00853223
EHD1	1.3741294	1.06834063	1.7674434	0.01333474
FAM198B	1.1549562	1.03224683	1.2922527	0.01194515
PBK	0.8716178	0.78451066	0.9683967	0.0105352
ZBTB47	1.3891018	1.13483005	1.7003461	0.00144184
RILPL2	1.236657	1.01536142	1.5061834	0.03472892
ULK4	0.5787046	0.39995626	0.837339	0.00371038
IL17RB	0.7956139	0.701598	0.9022282	0.00036587
KDF1	0.6640361	0.51704618	0.8528135	0.00134052
NID1	1.1924592	1.01953389	1.3947146	0.02766699
CLIC4	1.2036003	1.05677744	1.3708219	0.00523894
ARHGAP22	1.2984484	1.00470679	1.67807	0.04595039
SUOX	0.7530191	0.57596553	0.9844995	0.03806133
STRA6	1.202709	1.06038559	1.3641348	0.00407346
PFDN2	1.4243249	1.10009097	1.8441214	0.00727939
NOSIP	0.7613825	0.60740346	0.9543958	0.01803653
CLEC11A	1.2875191	1.10587784	1.4989951	0.00112598
KDM6B	1.3764675	1.08817927	1.741131	0.00770518
PKM	1.3015142	1.0817938	1.5658614	0.00521705
PTPRU	1.2825805	1.14127315	1.4413838	2.93E-05
GRPEL1	0.7713355	0.59645809	0.9974857	0.04779858
AFAP1-AS1	1.1162501	1.03053181	1.2090984	0.00698182
IKBIP	1.1957665	1.00703757	1.4198652	0.0413521
RFTN2	1.538635	1.11961544	2.114474	0.00789483
MESP2	1.3989256	1.08681246	1.8006721	0.00915325
KLHL41	0.5346164	0.29195611	0.9789648	0.04247428
DCBLD2	1.4666615	1.28436566	1.6748314	1.55E-08
PIGG	0.5883078	0.39678006	0.8722871	0.00829319
SYT1	1.1585304	1.01786867	1.3186305	0.02587136
DENND2A	0.7977597	0.65624246	0.9697949	0.0233414
MMAA	0.6805101	0.51472743	0.8996877	0.00689218
MAPK12	1.3950072	1.14345111	1.7019051	0.0010335
COPS7A	1.566688	1.13200501	2.1682867	0.00677372
SELE	1.1603481	1.0481971	1.2844986	0.00413611
SGPP2	0.8513713	0.75201109	0.9638595	0.01104318
TMEM63B	1.4020449	1.05721061	1.8593549	0.01896468
CCDC151	1.6492336	1.02776671	2.6464874	0.03812849
PSRC1	0.8112581	0.6846218	0.9613186	0.01571211
PLTP	1.118991	1.00958687	1.2402507	0.03221582
SPON1	1.0862365	1.00022728	1.1796416	0.04937218

MPC2	0.7237753	0.60056502	0.8722631	0.00068542
CMTM7	1.4018323	1.15184928	1.7060686	0.00074987
FAM171B	1.3225871	1.04687324	1.6709155	0.01907794
RPS17	1.4029548	1.02864654	1.9134679	0.03248905
SMPD2	0.744296	0.57071994	0.9706626	0.02927642
YPEL3	1.2204024	1.02641862	1.4510473	0.02412057
UNC5B	1.2182192	1.03375623	1.4355976	0.01845983
ANO1	1.1518062	1.04453435	1.2700948	0.00460399
RARB	1.3033772	1.01414415	1.6750993	0.03848286
H2AFZ	0.7511016	0.59791801	0.9435301	0.01391494
ZCCHC18	1.7343189	1.12509138	2.673438	0.01263904
STARD7-AS1	1.4487201	1.00851328	2.0810733	0.04487372
TREM2	1.1271148	1.00973664	1.2581377	0.03295299
B3GALT1	0.7339577	0.57323784	0.9397388	0.01417277
GPX3	1.1512813	1.04620426	1.2669119	0.00391458
TDRD7	0.7349282	0.56957049	0.9482926	0.01787425
B3GALNT1	1.1516202	1.00476729	1.3199366	0.04252998
AKT3	1.3625665	1.1494359	1.6152162	0.00036425
RGS3	1.5866838	1.12800223	2.23188	0.00800494
SIN3B	1.7856962	1.27721706	2.4966084	0.00069642
MCCC1	0.7787957	0.61237772	0.9904388	0.04152087
DDIT3	1.2614167	1.05506079	1.5081331	0.01083323
SLC35G2	1.3363723	1.07542441	1.6606383	0.00889665
CYMP	0.4676177	0.24816578	0.8811299	0.01869989
NPC2	1.2903226	1.01133492	1.646272	0.04030183
PMM1	0.7078364	0.57832231	0.8663548	0.00080412
ARRB1	0.7300781	0.58033937	0.9184524	0.00722457
ZFP37	1.3792635	1.06614164	1.7843482	0.01438728
C4orf19	0.7775726	0.68239491	0.8860254	0.00015909
TEX11	0.6395138	0.4765885	0.8581364	0.00288528
HOXB2	1.2237448	1.0846514	1.3806754	0.0010383
FZD9	0.8040774	0.65775975	0.9829433	0.03335033
FDFT1	0.7437235	0.62072222	0.8910986	0.00132746
PAQR6	1.3762495	1.14118532	1.6597329	0.00083178
BSPRY	0.8255083	0.68645875	0.9927237	0.04159367
AKAP5	0.7854711	0.63461976	0.9721803	0.02646856
ADORA2B	0.8659769	0.76256426	0.9834136	0.02657273
BEND3	0.6773746	0.51518267	0.8906285	0.00528061
ITGA3	1.2017388	1.01085059	1.4286743	0.0373199
LSM3	0.7303319	0.5507658	0.9684419	0.02905896
NR2F1	1.1476752	1.02103173	1.2900268	0.02095164
NLGN2	1.2635062	1.04812385	1.5231482	0.01417149
RING1	1.5169115	1.09514311	2.1011139	0.01218539
OIT3	0.7323451	0.60175274	0.8912786	0.00188007
EFNB2	1.2269442	1.06117052	1.4186147	0.005751
KCNK10	0.6914563	0.49160473	0.9725534	0.03401738
KLK10	1.1368202	1.05813693	1.2213543	0.00045807
TTC7B	1.2420598	1.05035878	1.4687482	0.0112643
LETM1	0.6272545	0.46154509	0.8524589	0.00288401
RTCB	0.6191461	0.46195054	0.8298333	0.0013356
ZNF385C	1.518627	1.14004225	2.0229319	0.00429238
PTPN14	1.6974905	1.403969	2.0523771	4.68E-08
PRPSAP1	0.6908161	0.47797804	0.9984286	0.04902976
SNAI1	1.4707959	1.21309813	1.7832362	8.65E-05

WASF1	1.2127595	1.06313952	1.3834362	0.00408748
LUM	1.0994531	1.00108424	1.2074879	0.04740942
KCNJ4	0.5983955	0.3796514	0.9431737	0.02696798
ADSL	0.7640875	0.60327418	0.9677685	0.02563511
ADH1A	0.6427077	0.45487072	0.9081112	0.012194
FES	1.3215646	1.08955851	1.6029732	0.00464305
FZD5	0.7497662	0.59927361	0.9380512	0.01175499
ALDH1A3	1.2350173	1.0535001	1.4478097	0.0092534
OR7E156P	1.6248724	1.05686009	2.4981645	0.02696938
QKI	1.2492812	1.06101572	1.4709524	0.0075708
PAFAH2	0.7805828	0.63016763	0.9669006	0.02331924
CD276	1.4708974	1.13781534	1.9014852	0.00322424
RIPK4	1.3425232	1.06864048	1.6865995	0.01139839
SLC23A2	1.2518692	1.00094656	1.5656944	0.04903885
LY6D	1.149085	1.03816713	1.2718533	0.00729238
INHBB	1.3304119	1.21588414	1.4557274	5.10E-10
KAT6B	1.3464388	1.01546548	1.7852872	0.03877318
TIMP4	0.8598088	0.74369164	0.9940561	0.04130051
NUBP1	0.7310215	0.53577003	0.9974288	0.0481318
EPHB2	0.7500556	0.64975904	0.865834	8.60E-05
AKAP12	1.157164	1.05176664	1.2731233	0.0027374
ENG	1.3725835	1.15271949	1.6343833	0.00037707
LINC00520	1.2206065	1.01461995	1.4684122	0.03452633
PLOD1	1.2955598	1.05387793	1.5926657	0.01396667
NOTCH2	1.4340526	1.16087161	1.7715196	0.00082753
TUFM	0.7223326	0.52915057	0.9860413	0.04051176
RHBDF2	1.2637566	1.04836961	1.5233948	0.01407116
CCL20	0.9218013	0.8572576	0.9912045	0.02791395
ARHGEF2	1.7196925	1.30520792	2.2658016	0.00011669
CERCAM	1.3786406	1.20131793	1.5821373	4.85E-06
FBXW9	0.7771369	0.6067517	0.9953688	0.04585513
PEAR1	1.3348371	1.09508839	1.6270743	0.00424657
BDNF	1.4951248	1.06644003	2.0961312	0.01964288
NR3C2	0.879082	0.79822544	0.9681289	0.00884704
WDR66	1.5799046	1.03748813	2.4059056	0.03304991
CASP3	0.7432235	0.60308952	0.9159191	0.00537157
CTDSP1	1.5373866	1.10843195	2.1323435	0.00997365
RFPL3S	1.6765562	1.00153003	2.8065465	0.04932402
JDP2	1.4370412	1.10145028	1.8748803	0.00753881
APBB2	1.2433725	1.0013701	1.54386	0.04856837
GEM	1.1767325	1.04940864	1.3195044	0.00534642
LAMA5	1.1925334	1.05545372	1.3474167	0.00470975
NPL	1.3508084	1.11160383	1.641487	0.00249491
LGALS1	1.1539277	1.02233065	1.3024644	0.02047969
CHAF1A	0.76262	0.59639807	0.9751695	0.03074055
SMARCA1	1.1715059	1.03205586	1.3297982	0.0143683
SLC26A3	0.9509984	0.90729412	0.996808	0.03633488
HSD11B2	0.8681605	0.78529563	0.9597693	0.00574073
PODXL	1.2163549	1.04508886	1.4156875	0.01142096
MAPK11	1.2996945	1.06297172	1.5891352	0.01061064
SLC12A6	1.4045209	1.08141422	1.8241658	0.01087263
FAM69C	1.4767634	1.1182836	1.9501585	0.00599635
FGF19	1.1782856	1.07990071	1.2856339	0.00022612
PODN	1.227187	1.06118032	1.419163	0.00576715

COL6A3	1.1202651	1.016131	1.235071	0.02252261
PLK3	1.6110906	1.23391833	2.1035532	0.00045733
DAO	0.5367938	0.31911493	0.9029587	0.01904403
MCC	1.3733088	1.14358296	1.6491825	0.00068262
NFATC4	1.4311734	1.14201317	1.7935497	0.00185123
KRT81	1.2916968	1.09153385	1.5285651	0.00288724
GIF	0.871535	0.76901358	0.9877241	0.03128653
OGFOD3	0.7346463	0.5573268	0.9683819	0.02867512
ADAMTS18	2.1264955	1.4587243	3.0999574	8.73E-05
COA3	0.6626424	0.49781931	0.8820367	0.0047998
POLR2I	0.7585904	0.60775042	0.9468679	0.01458076
CLK3	1.634609	1.10541672	2.4171395	0.01381202
LILRA2	1.3660161	1.00581987	1.8552029	0.04581201
SLC6A17	1.7186466	1.10745999	2.6671356	0.01572754
CDCA2	0.8232002	0.70821024	0.9568608	0.01126389
TMEM178A	1.2269011	1.01337585	1.4854177	0.03606748
MRPS18C	0.4870948	0.3242792	0.7316577	0.00053001
FLOT1	1.9688397	1.43686886	2.6977618	2.49E-05
DNAJB4	1.2064749	1.03369764	1.4081309	0.01730146
PXMP2	0.6492979	0.53089295	0.7941107	2.62E-05
STPG2	2.2704592	1.18202358	4.3611524	0.01381325
MYO5B	0.6979845	0.5752954	0.8468386	0.00026688
DLX2	1.3909678	1.03068536	1.8771893	0.03096117
ARPC2	1.516996	1.00225426	2.2961009	0.0487683
SLC41A3	1.4485166	1.08889388	1.9269099	0.01093227
SMARCD3	1.5641659	1.22663513	1.9945742	0.00030969
C3orf18	1.3784358	1.09248782	1.7392279	0.00681642
TRIB2	1.1856481	1.05586689	1.3313813	0.00398869
RFT1	0.7034128	0.50503821	0.9797071	0.03741124
MORF4L1	1.3807701	1.09884791	1.7350227	0.00562409
AP2M1	1.6805774	1.21568757	2.3232454	0.00167755
SYDE1	1.3194564	1.09194891	1.5943651	0.0040926
EID1	1.2825027	1.06280352	1.5476173	0.0094508
NOS2	0.8618052	0.78673419	0.9440396	0.00138197
THOP1	0.7428302	0.59697622	0.9243194	0.00768517
DUSP22	1.410842	1.10030531	1.8090207	0.00665603
GPRIN3	0.8339494	0.70639462	0.984537	0.03203576
CRISPLD1	1.2021992	1.06050865	1.3628204	0.00400003
ASPN	1.101322	1.02401546	1.1844647	0.00934796
MAPKBP1	1.7633289	1.2996112	2.392507	0.00026921
GJA1	1.2223582	1.08381038	1.3786172	0.00107082
PPL	1.1788279	1.0288196	1.3507082	0.01783148
PIP5K1C	1.2801276	1.01124281	1.6205077	0.04008223
NPR1	1.2605504	1.01859461	1.5599802	0.03322154
ROMO1	0.8180053	0.68952802	0.9704213	0.02120121
MPDZ	1.2407506	1.06835777	1.4409611	0.00470896
AGRN	1.3074125	1.07587094	1.5887847	0.00703233
ANKRD22	0.8387922	0.74250725	0.9475628	0.00471676
MTMR9LP	1.3659303	1.0706449	1.742656	0.01209916
ERI1	0.8193313	0.67708783	0.9914574	0.04054579
PLVAP	1.1715643	1.00257564	1.3690367	0.04633479
GUCY1A2	1.3500313	1.09157872	1.6696775	0.00563754
LGALS14	1.3627348	1.03618764	1.7921909	0.02680837
DOK3	1.2485681	1.02824954	1.5160932	0.02501264

BLNK	0.8515731	0.75821286	0.956429	0.00669019
PLA1A	1.1327987	1.02102305	1.2568108	0.01864855
CPT2	0.730916	0.58712524	0.9099219	0.0050388
STC1	1.1556906	1.02841178	1.2987219	0.01507574
ARC	1.3189417	1.12851949	1.5414951	0.00050185
SELENBP1	0.9003423	0.83075106	0.975763	0.01053519
DDX39A	0.7690038	0.6158611	0.9602275	0.02044087
SBNO2	1.262353	1.0011684	1.5916754	0.04885705
PDSS2	0.7233637	0.56436143	0.9271631	0.01055435
CPN1	0.8368462	0.704143	0.9945588	0.04318672
ERGIC3	0.7033436	0.5358431	0.9232035	0.01122117
SNAP23	1.2903943	1.03883824	1.6028651	0.02120244
ZDBF2	1.3021358	1.04500116	1.6225415	0.01866581
ZDHHC3	0.6193168	0.45380178	0.8452	0.00252759
MRPS25	0.6762188	0.50230146	0.9103535	0.00990533
RRAGD	1.1402579	1.00585019	1.2926261	0.04025538
MCAT	0.7341879	0.57974854	0.9297684	0.0103387
CDC42EP4	0.7305144	0.54996017	0.9703452	0.03017491
THBS3	1.5193693	1.25679946	1.836795	1.55E-05
COX10	0.6790209	0.51079319	0.9026536	0.00769741
LYPD6	0.7095572	0.58456206	0.8612796	0.00051964
HSPB1	1.1697465	1.01921062	1.3425163	0.02570158
PNLIPRP2	0.9152872	0.84069403	0.9964988	0.04126794
SLC22A17	1.2888124	1.04484123	1.589751	0.01780526
FAM166A	0.4978493	0.26552381	0.9334527	0.02965363
HOXC6	1.1567769	1.08037826	1.2385781	2.95E-05
ZNF135	1.564988	1.00396103	2.4395243	0.04799443
CCL22	0.7197148	0.61178005	0.8466923	7.27E-05
CSF1	1.2313822	1.00603508	1.5072059	0.04355895
SHPK	0.7556745	0.5722841	0.997833	0.04823867
LYPD3	1.1776862	1.02297434	1.3557961	0.02284185
SOX18	1.206611	1.01844424	1.4295432	0.02991254
